# Time-restricted feeding ameliorates non-alcoholic fatty liver disease through modulating hepatic nicotinamide metabolism via gut microbiota remodeling

**DOI:** 10.1080/19490976.2024.2390164

**Published:** 2024-08-18

**Authors:** Ruijia Feng, Wenchao Yang, Weiqi Feng, Xiuyi Huang, Meifeng Cen, Guiyan Peng, Wenrui Wu, Zhecun Wang, Yexiang Jing, Ting Long, Yunchong Liu, Zilun Li, Guangqi Chang, Kan Huang

**Affiliations:** a Division of Vascular Surgery, The First Affiliated Hospital, Sun Yat-sen University, Guangzhou, China; b National-Guangdong Joint Engineering Laboratory for Diagnosis and Treatment of Vascular Diseases, The First Affiliated Hospital, Sun Yat-sen University, Guangzhou, China; c Guangdong Provincial Key Laboratory of Malignant Tumor Epigenetics and Gene Regulation, Guangdong-Hong Kong Joint Laboratory for RNA Medicine, Medical Research Center, Sun Yat-Sen Memorial Hospital, Sun Yat-sen University, Guangzhou, China; d Organ Transplant Center, The First Affiliated Hospital, Sun Yat-sen University, Guangzhou, China

**Keywords:** Non-alcoholic fatty liver disease, time-restricted feeding, nicotinamide metabolism, gut microbiota

## Abstract

Non-alcoholic fatty liver disease (NAFLD) has emerged as a global health concern, lacking specific therapeutic strategies. Time-restricted feeding (TRF) regimen demonstrated beneficial effects in NAFLD; however, the underlying mechanisms remain unclear. In this study, we established a NAFLD mouse model through a high-fat diet (HFD) and implemented the 16:8 TRF regimen for a duration of 6 weeks. We demonstrated that TRF remarkably alleviated hepatic steatosis in HFD mice. Of note, aldehyde oxidase 1 (AOX1), a key enzyme in hepatic nicotinamide (NAM) catabolism, exhibited apparent upregulation in response to HFD, leading to abnormal accumulation of N-Methyl-6-pyridone-3-carboxamide (N-Me-6-PY, also known as 2PY) and N-Methyl-4-pyridone-5-carboxamide (N-Me-4-PY, also known as 4PY), whereas it was almost restored by TRF. Both N-Me-6-PY and N-Me-4-PY promoted de novo lipogenesis and fatty acid uptake capacities in hepatocyte, and aggravated hepatic steatosis in mice either fed chow diet or HFD. In contrast, pharmacological inhibition of AOX1 was sufficient to ameliorate the hepatic steatosis and lipid metabolic dysregulation induced by HFD. Moreover, transplantation of fecal microbiota efficiently mimicked the modulatory effect of TRF on NAM metabolism, thus mitigating hepatic steatosis and lipid metabolic disturbance, suggesting a gut microbiota-dependent manner. In conclusion, our study reveals the intricate relationship between host NAM metabolic modification and gut microbiota remodeling during the amelioration of NAFLD by TRF, providing promising insights into the prevention and treatment of NAFLD.

## Introduction

Non-alcoholic fatty liver disease (NAFLD) is a prevalent metabolic disorder characterized by excessive accumulation of lipid in hepatocytes.^1^ The global incidence rate of NAFLD is currently estimated to range from 25% to 30%.^2^ Furthermore, given the sedentary lifestyle and overnutrition diet in modern society, its incidence will continuously increase in future.^3,4^ NAFLD encompasses a spectrum of diseases that progress chronically, starting from simple steatosis and advancing to nonalcoholic steatohepatitis, cirrhosis, and hepatocellular carcinoma.^5^ Additionally, NAFLD elevates the risk of cardiovascular disease, type 2 diabetes mellitus, and chronic kidney disease, posing a substantial threat to public health.^6–8^ However, to date, there remains no FDA-approved therapy specifically targeting NAFLD. Lifestyle management and complications prevention serve as cornerstones for NAFLD treatment.^9,10^

Intermittent fasting (IF), a popular dietary pattern, has received remarkable attention due to its notable beneficial effects on various metabolic disorders, cancer, and cardiovascular diseases.^11–14^ As one of the IF regimens, time-restricted feeding (TRF) refers to consuming food within a specific time window each day. The commonly utilized 16:8 model entails an 8-h eating period followed by a 16-h fasting period. Numerous clinical studies and animal experiments consistently demonstrate that TRF exhibits robust metabolic modulatory effects and efficaciously protects against the occurrence and progression of NAFLD.^15–17^ Previous research had proposed that beyond weight loss, there existed additional insights that contribute to cardiometabolic health through TRF.^18^ Therefore, further elucidating the underlying mechanisms shall be a promising strategy for developing novel therapeutic targets on NAFLD.

Gut microbiota dysbiosis is a crucial pathophysiological mechanism of NAFLD.^19^ Patients with NAFLD display distinct intestinal bacterial profiles compared to healthy individuals, which are considered to accelerate hepatic steatosis, fibrosis, and inflammation.^20^ In fact, accumulating studies have documented that TRF markedly enhances the richness and diversity of intestinal microbiota, boosts the abundance of beneficial flora such as Faecalibacterium, Lactobacillus, Bacteroidetes, and Akkermansia, and restores normal microbial diurnal oscillations.^21,22^ Interestingly, a recent study provided initial evidence that transplantation of TRF microbiota could ameliorate nonalcoholic steatohepatitis in mice,^23^ suggesting a causative relationship between the reshaping of gut microbiota and the metabolic benefits of TRF pattern. However, the underlying mechanistic insights remain completely elusive.

Herein, we developed a NAFLD mouse model using a high fat diet (HFD) and provided the traditional 16:8 TRF regimen for a duration of 6 weeks. Our findings revealed that TRF exerted multifactorial alleviating effects in NAFLD. Mechanistically, TRF downregulated the expression of aldehyde oxidase 1 (AOX1), a key enzyme in hepatic nicotinamide (NAM) metabolism, which suppressed the overproduction of pro-steatotic substances N-Methyl-6-pyridone-3-carboxamide (N-Me-6-PY, also known as 2PY) and N-Methyl-4-pyridone-5-carboxamide (N-Me-4-PY, also known as 4PY) that induced by HFD, thereby restoring the abnormal hepatic de novo lipogenesis and fatty acid uptake capacities. Notably, transplantation of fecal microbiota was sufficient to mimic the modulatory effect of TRF on NAM metabolism, thus ameliorating hepatic steatosis and lipid metabolic disturbance. Collectively, the present study provides mechanistic insights into the protective effects of TRF on NAFLD.

## Materials and methods

### Animal experiment and diet design

Five-to-six weeks old male C57BL/6J mice were obtained from the Animal Center of the First Affiliated Hospital of Sun Yat-sen University. The mice were housed in a specific-pathogen-free (SPF) environment under a 12-h light/dark cycle at a temperature of 25 ± 2°C and a humidity of 55–60%. Following a week of acclimatization, the mice were randomly divided into four groups and subjected to different dietary interventions for a duration of 12 weeks (*n* = 8 per group): (1) chow diet (CD); (2) HFD containing 60% fat (TROPHIC, TP23400); (3) TRF: HFD for the initial 6 weeks, followed by a 16:8 regimen consisting of an 8-h eating period and a 16-h fasting period, with ad libitum access to water; (4) pair feeding (PF): HFD for the initial 6 weeks, with the average daily intake matching that of the TRF group but without time window restriction for another 6 weeks. Throughout the intervention, daily food intake was recorded, and body weight was measured weekly. Fresh fecal samples were collected under sterile environment during the last 3 days of week 12 for subsequent microbiota transplantation. At the end of the 12-week period, the mice were anesthetized with 1% sodium pentobarbital (100 mg/kg) via intraperitoneal injection after a 4-h fast. Blood samples were immediately collected from the retro-orbital plexus into EDTA-K2-coated microtubes. Plasma was obtained by centrifugation at 3000 rpm for 15 min at 4°C. The liver and epididymal adipose tissue was excised and weighed, which was fixed by 4% paraformaldehyde or cryopreserved at −80°C. The liver index was determined by dividing the liver weight by the body weight. Cecal contents were also collected and cryopreserved at −80°C for metagenome sequencing analysis. All animal experiments were approved by the Institutional Animal Care and Use Committees of the First Affiliated Hospital of Sun Yat-sen University and complied with the National Institutes of Health Guidelines on the Care and Use of Laboratory Animals.

### Histological evaluation

The left lateral lobe of the liver and epididymal adipose tissues fixed by 4% paraformaldehyde for 24 h were subsequently embedded in paraffin. Sections with a thickness of 4 µm were stained using the hematoxylin and eosin (H&E) method following standard protocols. Representative images were captured by an optical microscope at 200× magnification. The NAFLD activity score (NAS) was determined based on the steatosis grade (0–3), lobular inflammation (0–3), and hepatocyte ballooning (0–2), which collectively reflected the progression and severity of NAFLD.^24^ For oil red O staining, the liver tissues were embedded in an optimal cutting temperature compound and frozen sectioned. The oil red O positive areas and the size of epididymal adipocytes were calculated using ImageJ software (National Institutes of Health, USA).

### Biochemical assays

The levels of total triglyceride (TG), total cholesterol (TC), low-density lipoprotein cholesterol (LDL-c), alanine aminotransferase (ALT), alanine aminotransferase (AST), and reduced glutathione (GSH) in plasma or liver were determined by commercial assay kits following the manufacturer’s protocol (Nanjing Jiancheng Bioengineering Institute, A110-1-1, A111-1-1, A113-1-1, C009-2-1, C010-2-1, A006-2-1). Liver malondialdehyde (MDA) levels were also determined using the corresponding assay kits (Solarbio, BC0025). To prepare the liver homogenates, livers were weighed and mixed with 9 times the volume of ice-cold anhydrous ethanol or saline. The mixture was mechanically homogenized at 60 Hz for 1 min and then centrifuged at 2500–3500 rpm for 10 min at 4°C.

### Untargeted plasma metabolomic analysis

The plasma samples (50 µL) stored at −80°C were thawed on ice and then mixed with 300 µL extraction solution (acetonitrile: methanol = 1:4 V/V) containing internal standards in a 2 mL microcentrifuge tube. The mixture was vortexed for 3 min and then centrifuged at 12,000 rpm for 10 min at 4°C. Two hundred microliter supernatant was collected and placed in a −20°C environment for 30 min, followed by centrifugation at 12,000 rpm for 3 min at 4°C. Subsequently, 180 µL supernatant was obtained for further analysis using ultra-performance liquid chromatography (UPLC, ExionLC AD, https://sciex.com.cn/) and tandem mass spectrometry (MS/MS, QTRAP® System, https://sciex.com/).

The UPLC conditions were as follows: (1) chromatographic column: Waters ACQUITY UPLC HSS T3 C18 (1.8 µm, 2.1 mm *100 mm); (2) mobile phase: 0.1% formic acid in water as phase A and 0.1% formic acid in acetonitrile as phase B; (3) gradient elution: 95:5 V/V at 0 min, 10:90 V/V at 11.0 min, 10:90 V/V at 12.0 min, 95:5 V/V at 12.1 min, 95:5 V/V at 14.0 min; (4) flow rate: 0.4 mL/min; (5) column temperature: 40°C; (6) sample size: 2 µL. The specific parameters of MS/MS were presented as follows: (1) electrospray ionization (ESI): 500°C; (2) voltage: 5500 V (positive), −4500 V (negative); (3) ion source gas: gas I (GSI), gas II (GSII), and curtain gas (CUR) were set at 55, 60, and 25 psi, respectively; (4) collision-activated dissociation (CAD): high. Each ion pair was detected based on optimized de-clustering potential and collision energy.

Metabolite qualitative profiling was performed using the MWDB database, while quantitative analysis was conducted using multiple reaction monitoring (MRM). The orthogonal partial least squares discriminant analysis (OPLS-DA) was carried out using R software (version 4.2.2; https://www.r-project.org) with the MetaboAnalyst R package. The differential metabolites were annotated and subsequently mapped to the KEGG Pathway database, which were then subjected to metabolite set enrichment analysis (MSEA) in MetaboAnalyst 5.0.

### Targeted liquid chromatography-mass spectrometry (LC-MS/MS)

Fifty microliter plasma samples stored at −80°C were thawed on ice and then combined with 200 µL ice-cold methanol containing internal standard. One hundred milligram liver samples were also thawed and mechanically homogenized with 1 mL ice-cold methanol. The above mixture was further vortexed and centrifuged at 14,000 rpm for 10 min at 4°C. The supernatant was collected and lyophilized by a freeze dryer. The lyophilized powder was dissolved in 50 µL 50% acetonitrile. Subsequently, the clear supernatant was transferred to glass vials with microinserts, and LC-MS analysis was conducted using Agilent 1290 Infinity II UPLC −6470B Triple-quadrupole LC-MS system with a dual AJS ESI source (Agilent Technologies, Inc., USA). The chromatographic settings were as follows: (1) chromatographic column: Thermo Hypersil Gold C18 column (1.9 µm, 2.1 mm *100 mm); (2) mobile phase: 0.1% formic acid in water as phase A and 0.1% formic acid in acetonitrile as phase B; (3) gradient elution: 0–0.5 min, 95:5 V/V, 0.5–3 min, 95:5 -V/V→5:95 V/V, 3–3.5 min, 5:95 V/V→95:5 V/V, equilibrate for 5 min; (4) flow rate: 0.3 mL/min; (5) sample size: 1 µL. MRM in positive electrospray ionization mode (Agilent Jet Stream) was utilized with the following transitions: N-Me-6-PY at m/z 152.9 → 107.9 and m/z 152.9 → 67.0; N-Me-4-PY at m/z 152.9 → 135.9 and m/z 152.9 → 92; 1-methylnicotinamide (1-MNA) at m/z 138 → 78.6 and m/z 138 → 94.6; NAD+ at m/z 664 → 428 and m/z 664 → 136; NAM at m/z 123 → 80 and m/z 123 → 53; nicotinic acid (NA) at m/z 124 → 80 and m/z 124 → 78. The ion source parameters were as follows: (1) gas temperature: 300°C; (2) gas flow: 5 L/min; (3) nebulizer: 45 psi; (4) sheath gas flow: 11 L/min; (5) capillary voltage: 3500 V (positive) and 3500 V (negative); (6) nozzle voltage: 500 V (positive) and 500 V (negative). All metabolites were quantified according to the standard curve running at the same batch of samples.

### Metagenome sequencing

The microbial DNA was extracted using Magnetic Soil and Stool DNA Kit (TIANGEN BIOTECH, DP712). The DNA degradation degree and potential contamination were monitored by 1% agarose gels. The DNA concentration was measured using Qubit® dsDNA Assay Kit on the Qubit® 2.0 Fluorometer (Life Technologies, CA, USA). One microgram DNA was used as input material for DNA sample preparation. Sequencing libraries were generated by the NEBNext® Ultra™ DNA Library Prep Kit for Illumina (NEB, USA) according to the manufacturer’s instructions. Index codes were subsequently added to assign sequences to each sample. The DNA sample was initially fragmented into approximately 350 bp length by sonication. Subsequently, the DNA fragments underwent end-polishing, A-tailing, and ligation with a full-length adaptor specifically designed for Illumina sequencing. PCR amplification was then conducted to enrich the DNA fragments. PCR products were purified using the AMPure XP system, and the libraries were assessed for size distribution using the Agilent 2100 Bioanalyzer and quantified using real-time PCR. The clustering of the index-coded samples was performed on a cBot Cluster Generation System according to the manufacturer’s instructions. Once cluster generation was complete, the library preparations were sequenced on an Illumina NovaSeq platform, which generated paired end (PE) reads.

The raw data underwent quality control using the Fastp software. The assembly analysis was conducted using the MEGAHIT Assembly software, with the following parameters: –k-min 35, –k-max 95, –k-step 20, –min-contig-len 500. Subsequently, the clean data was aligned to the contigs of each sample using the Bowtie2 software to identify PE reads that were not utilized. The comparison parameters used were: -I 200, -X 400. The unutilized reads from each sample were then combined for a mixed assembly, using the same assembly parameters as mentioned above. To predict Open Reading Frames (ORF), MetaGeneMark was applied to all contigs (≥500 bp). The predicted genes with a length less than 100nt were filtered out. CD-HIT software was used to remove the redundancy, so as to obtain a non-redundant initial gene catalog. By default, identity 95% and coverage 90% were used for clustering, and the longest sequence was selected as the representative sequence. Parameters: -c 0.95, -g 0, -AS 0.9, -g 1, -d 0. Then, Bowtie2 was used to compare the clean data of each sample to the initial gene catalog, and the number of reads that matched each gene was calculated. The comparison parameters used were as follows: –end-to-end, –sensitive, -I 200, -X 400. Genes with supporting reads ≤2 in each sample were filtered out to obtain a gene catalog (Unigenes) for subsequent analysis. Based on the number of reads and gene length compared, the abundance information of each gene in each sample was calculated.

To explore the taxonomy abundance information of each sample at each classification level (phylum, class, order, family, genus, and species), the Unigenes were compared with sequences of bacteria, fungi, archaea, and viruses extracted from the NCBI NR database via DIAMOND software and LCA algorithm of MEGAN software (blastp, evalue ≤1e-5). Relative abundance comparison, principal coordinate analysis (PCoA), cluster analysis, and Metastats multivariate analysis were performed based on the abundance tables at each classification level.

### Fecal microbiota transplantation (FMT)

Five-to-six weeks old male C57BL/6J mice fed a HFD for 9 weeks were set as recipients. The recipients were administered with an antibiotic cocktail consisting of 1 mg/mL neomycin (MCE, HY-B0470), 1 mg/mL streptomycin (MCE, HY-B1906) and 1 mg/mL bacitracin (APExBIO, B1670) in their drinking water for 1 week prior to FMT. Mice from HFD and TRF groups were selected as the donors for FMT. The feces of the donors were collected in a sterile environment and stored at −80°C. The feces were then mixed with sterile phosphate-buffered saline (PBS) (100 mg feces/1 ml PBS). The mixture was vortexed for 10 min and centrifuged at 1000 rpm for 5 min at 4°C. The supernatant was administrated to recipients (200 µL per mice) by oral gavage 3 times a week for 4 weeks.

### Cell culture

HepG2 cells were cultured in Dulbecco’s Modified Eagle Medium (DMEM) (Gibco, C11995500BT) supplemented with 10% fetal bovine serum (FBS) (Gibco 10,091,148). The cells were incubated at 37°C and with 5% carbon dioxide. N-Me-6-PY (MCE, HY-113432) and N-Me-4-PY (MCE, HY-113472) were dissolved in DMEM and stored at −20°C. Different concentrations of N-Me-6-PY, N-Me-4-PY or their vehicle were added to the cells 1 h prior to treatment. The cells were then treated with free fatty acids (FFA) consisting of oleic acid (OA) (Sigma-Aldrich 75,090) and palmitic acid (PA) (Solarbio, P9830) in a ratio of 2:1, at a total concentration of 0.6 mM for 24 h to establish an *in vitro* NAFLD model.

### Cell counting kit-8 (CCK8) assay

The cytotoxicity analysis was conducted by the CCK8 method (Beyotime, C0037). After HepG2 cells were seeded in a 96-well plate and cultured for 24 h, different concentrations of N-Me-6-PY or N-Me-4-PY were added and cultured for an additional 24 h. Then, 10 µL CCK8 reagent was added to each well and incubated for 1 h at 37°C. Following the incubation, the absorbance of each well was measured using a microplate reader at a wavelength of 450 nm. The median inhibitory concentration (IC50) was calculated by GraphPad Prism Version 9.0 software.

### Oil red O staining and TC, TG measurement

HepG2 cells seeded on a 24-well plate were fixed with 4% paraformaldehyde for 15 min at room temperature. After being rinsed by PBS, the cells were stained with filtered oil red O solution (Sigma-Aldrich, O0625) for 30 min at 37°C. Subsequently, the cells were destained with 60% isopropyl alcohol for 5 s. Nuclei were stained with hematoxylin (Solarbio, G4070) for 1 min. The oil red O positive areas, indicating lipid accumulation, were evaluated using ImageJ software. Intracellular TG and TC were measured following the manufacturer’s instructions (Nanjing Jiancheng Bioengineering Institute, A110-1-1, A111-1-1). In brief, cells were digested and resuspended in PBS. After adequate sonication, the levels of TG and TC were measured and normalized to the corresponding protein concentrations.

### N-Me-PYs in vivo assay

N-Me-PYs, consisting of N-Me-6-PY (150 µg/kg) and N-Me-4-PY (75 µg/kg) dissolved in sterile PBS, were administered via intraperitoneal injection. Hydralazine (HYD, Sigma-Aldrich, H1753), a potent AOX1 inhibitor, was also dissolved in sterile PBS and administered via oral gavage at a dose of 50 mg/kg.^25^ Five-to-six weeks old male C57BL/6J mice were randomly divided into the following five groups for a 6-week intervention: (1) CD; (2) CD + N-Me-PYs; (3) HFD; (4) HFD + N-Me-PYs; (5) HFD + HYD. Body weight, food intake, plasma, liver, and epididymal adipose tissue samples were collected and processed as described above.

### Western blotting

Proteins from cells and livers were extracted by RIPA buffer (Fdbio science, FD009) supplemented with protease inhibitor cocktail (CWBIO, CW2200S) and phosphatase inhibitor cocktails (CWBIO, CW2383S). Protein concentration was determined by BCA assay kit (Fdbio science, FD2001). A total of 40 µg protein was separated on 10% sodium dodecyl sulfate polyacrylamide gel electrophoresis (SDS-PAGE) gels and further transferred on 0.2 µm polyvinylidene difluoride (PVDF) membrane. After being blocked by 5% skimmed milk for 1 h, the membrane was incubated with different primary antibodies overnight at 4°C. The primary antibodies against NNMT (15123–1-AP, 1:1000 dilution), AOX1 (19495–1-AP, 1:500 dilution), FASN (10624–2-AP, 1:1000 dilution), ACC1 (67373–1-Ig, 1:1000 dilution), FABP4 (12802–1-AP, 1:1000 dilution), FATP2 (14048–1-AP, 1:500 dilution) and β-actin (66009–1-Ig, 1:5000 dilution) were acquired from Proteintech, CD36 (YT5585, 1:1000 dilution) and SREBP-1c (YT6055, 1:1000 dilution) were obtained from Immunoway. The membrane was then rinsed with tris-buffered saline with 0.1% Tween-20 (TBS-T) and incubated with corresponding secondary antibodies (Proteintech, SA00001–1; Asbio, AS006; 1:5000 dilution) at room temperature for 1 h. The protein bands were visualized using electrochemiluminescence (ECL) reagents (Millipore, WBKLS0500) according to the manufacturer’s instructions. The relative band density was quantified by ImageJ software, with β-actin as an internal standard.

### Quantitative real-time reverse transcription polymerase chain reaction (qRT-PCR)

Total RNA was extracted using the AG RNAex Pro Reagent (Accurate Biotechnology, AG21102) following the manufacturer’s instructions. The concentration and purity of RNA were measured using a NanoDrop 2000 spectrophotometer. Subsequently, cDNA was obtained using the Evo M‐MLV RT kit (Accurate Biotechnology, AG11728). The primer sequences utilized in this study were obtained from PrimerBank, synthesized in GENEray Biotechnology, and listed in Table 1. qRT-PCR was conducted in a 10 μL reaction volume consisting of 1 μL cDNA, 0.5 μL specific forward/reverse primers, 5 μL SYBR Green Premix Pro Taq HS qPCR Kit (Accurate Biotechnology, AG11702), and 3 μL RNase-Free water on the LightCycler 480 instrument. The relative expression of different genes was calculated by the 2^−∆∆Ct^ method and normalized to GAPDH.

**Table 1.** Primer sequences information.

Species	Gene name	Primers
Mouse	SREBP-1c	5′-CAAGGCCATCGACTACATCCG-3′
		5′-CACCACTTCGGGTTTCATGC-3′
	FASN	5′-GGAGGTGGTGATAGCCGGTAT-3′
		5′-TGGGTAATCCATAGAGCCCAG-3′
	ACC1	5′-AATGAACGTGCAATCCGATTTG-3′
		5′-ACTCCACATTTGCGTAATTGTTG-3′
	CD36	5′-ATGGGCTGTGATCGGAACTG-3′
		5′-TTTGCCACGTCATCTGGGTTT-3′
	FATP2	5′-GATGCCGTGTCCGTCTTTTAC-3′
		5′-GACTTCAGACCTCCACGACTC-3′
	FABP4	5′-ATCAGCGTAAATGGGGATTTGG-3′
		5′-GTCTGCGGTGATTTCATCGAA-3′
	TNFα	5′-CTGAACTTCGGGGTGATCGG-3′
		5′-GGCTTGTCACTCGAATTTTGAGA-3′
	IL-6	5′-CTGCAAGAGACTTCCATCCAG-3′
		5′-AGTGGTATAGACAGGTCTGTTGG-3′
	IL-1β	5′-GAAATGCCACCTTTTGACAGTG-3′
		5′-TGGATGCTCTCATCAGGACAG-3′
	CPT1α	5′-AGATCAATCGGACCCTAGACAC-3′
		5′-CAGCGAGTAGCGCATAGTCA-3′
	ACOX1	5′-GAGCAGCAGGAGCGTTTCTT-3′
		5′-CAGGACTATCGCATGATTGGAAG-3′
	PPARα	5′-ACCACTACGGAGTTCACGCATG-3′
		5′-GAATCTTGCAGCTCCGATCACAC-3′
	NNMT	5′-GCCCCACCATCTATCAGCTTC-3′
		5′-ACGCCTCAACTTCTCCTCCT-3′
	AOX1	5′-AGGCCCCTATTGTCATGGG-3′
		5′-GAGCACGGTATGTCTGTGTCT-3′
	GAPDH	5′-TGGCCTTCCGTGTTCCTAC-3′
		5′-GAGTTGCTGTTGAAGTCGCA-3′
Human	SREBP-1c	5′-ACAGTGACTTCCCTGGCCTAT-3′
		5′-GCATGGACGGGTACATCTTCAA-3′
	FASN	5′-ACAGCGGGGAATGGGTACT-3′
		5′-GACTGGTACAACGAGCGGAT-3′
	ACC1	5′-ATGTCTGGCTTGCACCTAGTA-3′
		5′-CCCCAAAGCGAGTAACAAATTCT-3′
	CD36	5′-AAGCCAGGTATTGCAGTTCTTT-3′
		5′-GCATTTGCTGATGTCTAGCACA-3′
	FATP2	5′-TACTCTTGCCTTGCGGACTAA-3′
		5′-CCGAAGCAGTTCACCGATATAC-3′
	FABP4	5′-ACTGGGCCAGGAATTTGACG-3′
		5′-CTCGTGGAAGTGACGCCTT-3′
	TNFα	5′-GAGGCCAAGCCCTGGTATG-3′
		5′-CGGGCCGATTGATCTCAGC-3′
	IL-6	5′-CCTGAACCTTCCAAAGATGGC-3′
		5′-TTCACCAGGCAAGTCTCCTCA-3′
	IL-1β	5′-AGCTACGAATCTCCGACCAC-3′
		5′-CGTTATCCCATGTGTCGAAGAA-3′
	CPT1α	5′-ATCAATCGGACTCTGGAAACGG-3′
		5′-TCAGGGAGTAGCGCATGGT-3′
	ACOX1	5′-GGAACTCACCTTCGAGGCTTG-3′
		5′-TTCCCCTTAGTGATGAGCTGG-3′
	PPARα	5′-TTCGCAATCCATCGGCGAG-3′
		5′-CCACAGGATAAGTCACCGAGG-3′
	GAPDH	5′-CTGGGCTACACTGAGCACC-3′
		5′-AAGTGGTCGTTGAGGGCAATG-3′

### Statistical analysis

All data were presented as mean ± standard error of the mean (SEM). The normal distribution of the data was initially assessed by the Shapiro–Wilk test. Statistical comparisons between the two groups were conducted using the unpaired two-tailed Student’s t-test for normally distributed data, and the Welch’s t-test for non-normally distributed data. Differences among three or more groups were analyzed using one-way analysis of variance (ANOVA) with Fisher’s LSD post hoc test for normally distributed data, and the Welch’s ANOVA for non-normally distributed data. The non-parametric *Spearman* test was employed to evaluate correlations between quantitative data. Statistical analyses in this study were performed using GraphPad Prism Version 9.0 software, R Studio, and MetaboAnalyst 5.0. *p* value < 0.05 was considered statistically significant.

## Result

### TRF alleviates hepatic steatosis, not solely dependent on the reduction of food intake and body weight

**Figure 1.**
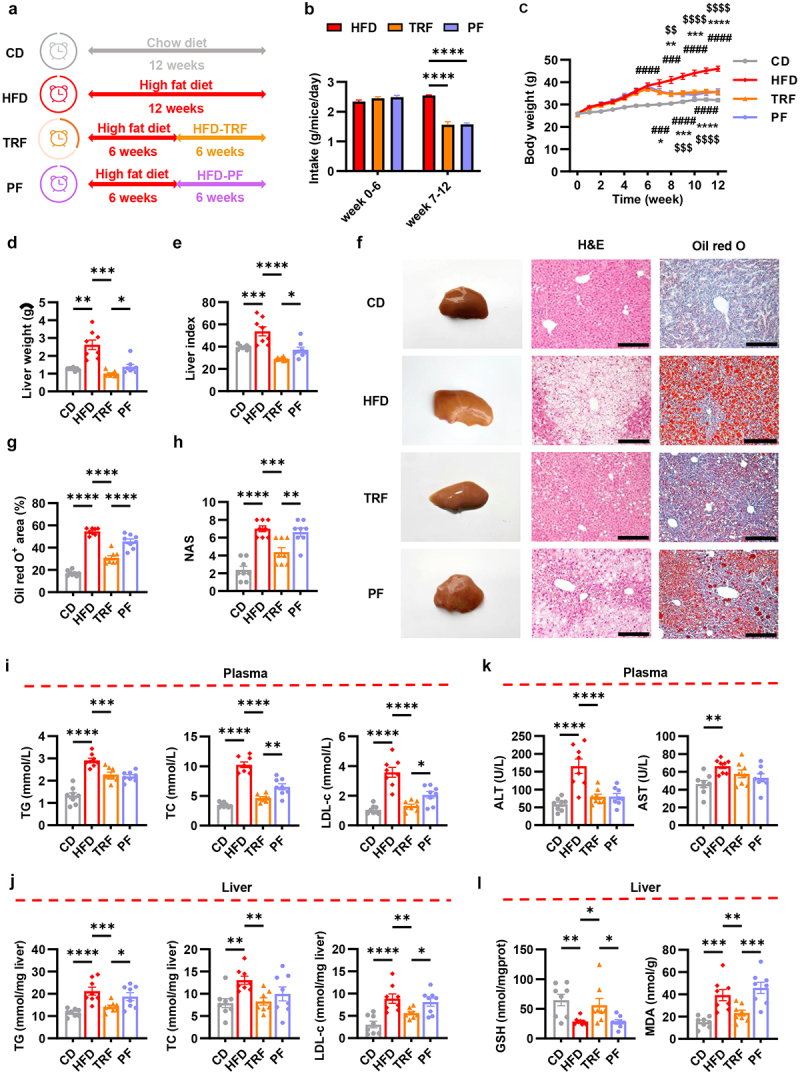
TRF alleviates hepatic steatosis, not solely dependent on the reduction of food intake and body weight. (a) The schematic diagram of animal experiment design. The proportion of clock color occupancy indicates the feeding time, with the TRF regimen involving unrestricted food intake in 8 hours each day. (b) Average food intake during the 12 weeks experimental period. (c) Body weight changes during the 12 weeks experimental period. (d-e) Mice liver weight (d) and liver index (e). (f) Representative macroscopic images and histological results of H&E staining and oil red O staining of mice liver. Scale bar = 200 μm. (g) Oil red O positive area quantification. (h) NAS quantification. (i-j) TG, TC, and LDL-c levels in plasma (i) and liver homogenates (j). (k) ALT and AST levels in plasma. (l) GSH and MDA levels in liver homogenates. *n* = 8 per group.

HFD was utilized to establish a mouse model of NAFLD, with standard CD serving as the control. After a duration of 6 weeks, mice fed a HFD were randomly divided into three groups – HFD ad libitum, TRF, or PF. The TRF regimen involved unrestricted food intake from 8:00 to 16:00 each day, with only water allowed during the remaining 16 h. The PF group consumed a similar amount of food per day as the TRF group, but without any restrictions on the time window for food intake (Figure 1(a)). During the 6-week intervention, TRF resulted in a 35% reduction in daily food consumption, accompanied by a 20% decrease in body weight (Figures 1(b–c) and S1A). Likewise, a comparable weight loss was observed in PF mice.

Data are presented as the mean ± SEM and further analyzed by Welch’s ANOVA or one-way ANOVA with Fisher’s LSD post hoc test. *P < 0.05; **P < 0.01; ***P < 0.001; ****P < 0.0001. In **(C)**, “#” denoted the comparison between CD and HFD, ”*” represented the comparison between HFD and TRF, and ”$” signified the comparison between HFD and PF. CD: chow diet; HFD: high fat diet; TRF: time restricted feeding; PF: pair feeding. NAS: NAFLD activity score; H&E: hematoxylin and eosin; TG: total triglyceride; TC: total cholesterol; LDL-c: low-density lipoprotein cholesterol; ALT: alanine aminotransferase; AST: alanine aminotransferase; GSH: reduced glutathione; MDA: malondialdehyde.

We then explored the impacts of different dietary patterns on liver steatosis. As expected, 12-week HFD led to apparent elevations in liver weight and liver index (Figures 1(d–e)), accompanied by the presence of yellow dot-like structures on the liver surface indicating lipid deposition (Figure 1(f)). Histological examination revealed augmented hepatic vacuolation, steatosis, as well as inflammatory cell infiltration (Figure 1(f)). Oil red O staining also confirmed a notable increase in lipid accumulation, and the NAS was markedly elevated (Figures 1(f–h)). Noteworthily, a six-week TRF resulted in an approximate 50% reduction in liver weight, liver index, and lipid deposition area in the liver (Figures 1(d–h)). The extent of steatosis, lobular inflammation, and hepatocyte ballooning, which constitute the three components of NAS, were all mitigated to some extent (Figure S1B), indicating a substantial improvement in NAFLD under the intervention of TRF. To further determine the impact of TRF on lipid accumulation, epididymal adipose tissue size was measured. Similarly, the enlargement of adipocytes induced by HFD was declined following TRF (Figure S1C), indicating that TRF ameliorated lipid overload in the organism. In contrast, although food intake restriction partially improved the aforementioned liver and adipose parameters, the protective effect of PF appeared to be weaker than that of TRF (Figures 1(d–h) and S1B-C). Supporting this, TRF exhibited a more pronounced effect, compared to PF, on modulating lipid homeostasis in both plasma and liver (Figures 1(i–j)). Moreover, while both TRF and PF displayed equivalent improvement on liver enzyme, as indicated by the decline of ALT (Figure 1(k)), TRF was sufficient to restore the hepatic GSH and MDA levels, which were unnoticeable in PF mice (Figure 1(l)). These data suggested a unique anti-oxidative effect of TRF. Taken together, our findings highly implicated that the alleviation of hepatic steatosis through TRF might not be solely attributed to decreased food intake and body weight.

### The reprogramming of NAM metabolism might mediate the beneficial effects of TRF on NAFLD improvement

**Figure 2.**
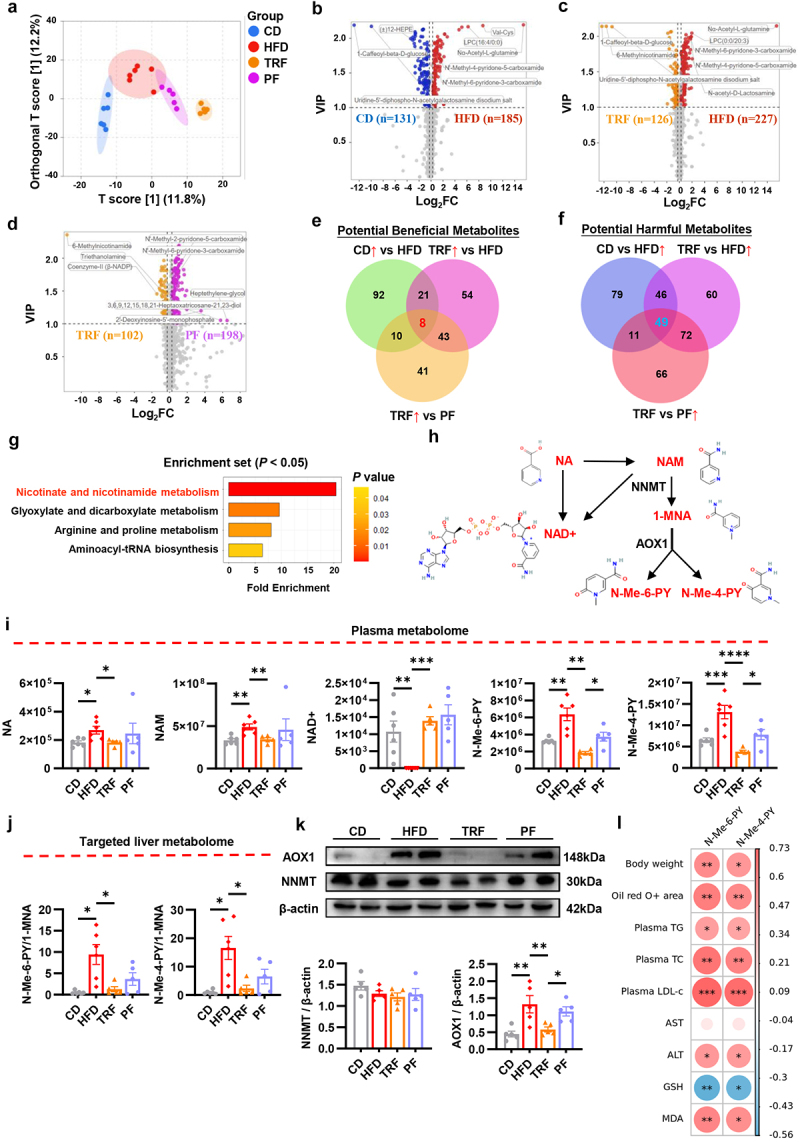
The reprogramming of NAM metabolism might mediate the beneficial effects of TRF on NAFLD improvement. (a) Orthogonal partial least squares discriminant analysis plot based on untargeted plasma metabolomic analysis (n = 6 in CD and HFD, n = 5 in TRF and PF). (b-d) Volcano plots showed differential metabolites (fold change > 1.2 or < 0.83 and VIP > 1) in CD vs HFD (b), HFD vs TRF (c) and TRF vs PF (d). (e-f) Overlap analysis identified potential beneficial (e) and harmful (f) metabolites mediated the improvement of NAFLD by TRF. (g) Metabolite set enrichment analysis based on the 57 potential metabolites that mediate the amelioration of NAFLD by TRF. All metabolic pathways with *P* < 0.05 were presented. (h) The overview of NAM metabolism pathway. (i) The plasma levels of NA, NAM, NAD+, N-Me-6-PY and *N*-Me-4-PY among four groups measured by metabolomic analysis (n = 6 in CD and HFD, n = 5 in TRF and PF). (j) The transversion ratio of N-Me-6-PY and N-Me-4-PY in mice liver (n = 6 in CD and HFD, n = 5 in TRF and PF). (k) Western blot analysis of NNMT and AOX1 in mice liver. β-actin was used as the internal standard (n = 5). (l) The heatmap showed the correlations between plasma N-Me-6-PY, N-Me-4-PY and various clinical parameters associated with NAFLD including body weight, oil red O staining positive area, plasma lipids, hepatic enzymes, and oxidative indicators. The color scale represents the *Spearman’s* correlation coefficient, and the circle size represents the significance (n = 6 in CD and HFD, n = 5 in TRF and PF).

Considering the robust association between NAFLD and organic metabolic dysfunction,^26,27^ along with the proven preservation of metabolic health by TRF,^27^ we conducted comprehensive untargeted metabolomic profiling of mice plasma samples to explore the metabolic alterations during dietary intervention. A total of 972 metabolites were identified in the analysis. OPLS-DA revealed an apparent disparity in the metabolite composition between TRF and HFD mice, which was less pronounced in PF versus HFD (Figure 2(a)). We then applied a combination of criteria including variable importance on projection (VIP) >1 and fold change (FC) >1.2 or <0.83 to identify the differential metabolites among various dietary patterns. Concretely, HFD led to an upregulation of 185 metabolites and the downregulation of 131 metabolites in comparison to CD (Figure 2(b)). However, upon dietary intervention with TRF, the plasma metabolites of HFD mice were significantly changed, with 126 metabolites elevated and 227 declined (Figure 2(c)). Of note, in spite of equal food intake, there remained 300 differential metabolites observed between TRF and PF mice (Figure 2(d)). The MSEA enrichment sets, which depicted the biologically meaningful contexts related to these altered metabolites,^28^ were shown in Figure S2A-C, respectively. These data further supported that the metabolic modulatory effect of TRF may not merely rely on food intake reduction.

Data are presented as the mean ± SEM and further analyzed by Welch’s ANOVA or one-way ANOVA with Fisher’s LSD post hoc test. The correlation was tested by nonparametric Spearman’s correlation test. *P < 0.05; **P < 0.01; ***P < 0.001; ****P < 0.0001. CD: chow diet; HFD: high fat diet; TRF: time restricted feeding; PF: pair feeding. VIP: variable importance on projection; NNMT: nicotinamide N-methyltransferase; AOX1: aldehyde oxidase 1; NA: nicotinic acid; NAM: nicotinamide; 1-MNA: 1-methylnicotinamide; N-Me-6-PY: N-Methyl-6-pyridone-3-carboxamide; N-Me-4-PY: N-Methyl-4-pyridone-5-carboxamide; TG: total triglyceride; TC: total cholesterol; LDL-c: low-density lipoprotein cholesterol; ALT: alanine aminotransferase; AST: alanine aminotransferase; GSH: reduced glutathione; MDA: malondialdehyde.

To investigate the particular regulatory effects of TRF on host metabolism, distinct from the effects of food restriction, we proceeded to identify the metabolites that were specifically influenced upon TRF intervention. As shown by the Venn diagrams, a total of 57 metabolites, including 8 potential beneficial (characterized as metabolites that were downregulated by HFD but restored by TRF rather than PF) and 49 potential harmful metabolites (opposite to beneficial metabolites), were searched out (Figures 2(e-f)). The relative abundances of these metabolites were depicted in Figure S2D. Using MSEA, these 57 differential metabolites were primarily involved in nicotinate (NA) and NAM metabolism, glyoxylate and dicarboxylate metabolism, arginine and proline metabolism, and aminoacyl-tRNA biosynthesis pathways (Figure 2(g)). Interestingly, the NA and NAM metabolism pathway ranked the top among them (Figure 2(g)). Consistent with this, the differential metabolites mediated by TRF were predominantly enriched in this pathway as well (Figure S2B). These findings highly announced the alteration of NA and NAM metabolism might be associated with the metabolic protective effect of TRF dietary pattern.

NA and NAM are two forms of vitamin B3 that act as precursors for the coenzymes nicotinamide adenine dinucleotide (NAD+) and nicotinamide adenine dinucleotide phosphate (NADP+), which were essential for maintaining energy and cellular homeostasis.^29^ On the other hand, NAM can be converted to 1-MNA by nicotinamide N-methyltransferase (NNMT) and subsequently metabolized to N-Me-6-PY and N-Me-4-PY by AOX1, predominantly in the liver^30^ (Figure 2(h)). Of note, the plasma levels of NA, NAM, N-Me-6-PY, and N-Me-4-PY were prominent increased in HFD mice, whereas NAD+ was almost completely exhausted (Figure 2(i)). Moreover, the conversions of 1-MNA to N-Me-6-PY and N-Me-4-PY in the liver were largely enhanced, as evidenced by the elevation of N-Me-6-PY/1-MNA and N-Me-4-PY/1-MNA ratios (Figures 2(j) and S2E). Supporting this, both the protein and gene expressions of hepatic AOX1, but not NNMT, were upregulated in HFD mice (Figures 2(k) and S2F). These observations demonstrated the augmentation of hepatic NAM catabolism in HFD mice. On the contrary, TRF intervention was capable of restoring the upregulation of hepatic AOX1 (Figures 2(k) and S2F) and the augmentation of hepatic NAM catabolism (Figures 2(j) and S2E) induced by HFD, thereby mitigating the overaccumulation of N-Me-6-PY and N-Me-4-PY in plasma (Figure 2(i)). While a mild ameliorating effect was also observed in PF mice, it seemed less pronounced compared to TRF (Figures 2(i–k) and S2E-F). In addition, both the plasma and the hepatic levels of NAD+ were largely increased upon TRF intervention (Figures 2(i) and S2E), suggesting an improvement of NAD+ metabolism. Intriguingly, the plasma levels of N-Me-6-PY and N-Me-4-PY presented dramatically positive correlations with various parameters that associated with hepatic steatosis and lipid metabolic disturbance, including body weight, oil red O positive area, plasma lipid parameters, ALT, and hepatic MDA, whereas displayed negative association with the hepatic GSH (Figure 2(l)), suggesting the potential pro-steatotic effect of N-Me-6-PY and N-Me-4-PY. Collectively, our findings indicated that TRF might alleviate NAFLD by suppressing the excessive accumulation of NAM metabolic end products, N-Me-6-PY and N-Me-4-PY.

### N-Me-6-PY and N-Me-4-PY promote hepatic de novo lipogenesis and fatty acid uptake, which exacerbate lipid deposition

**Figure 3.**
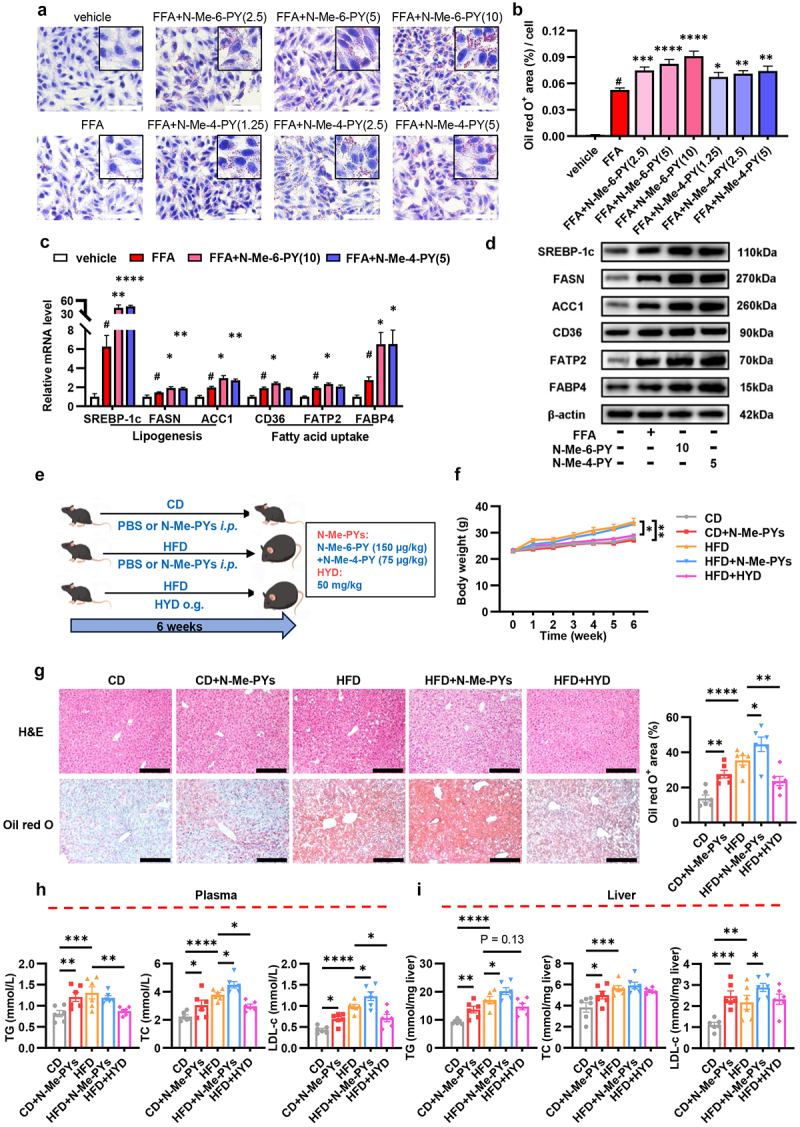
N-Me-6-PY and N-Me-4-PY promote hepatic de novo lipogenesis and fatty acid uptake, which exacerbate lipid deposition. (a–b) HepG2 cells were pre-treated with 2.5-fold, 5-fold, and 10-fold physiological concentrations of N-Me-6-PY or N-Me-4-PY for 1 hour and subsequently co-incubated with 0.6 mM FFA for 24 hours. Representative images of oil red O staining of HepG2 cells were presented (a) and quantified (b) (n = 5). Scale bar = 100 μm. (c) qPCR analysis of mRNA levels of genes associated with de novo lipogenesis (SREBP-1c, ACC1, FASN) and fatty acid uptake (CD36, FATP2, FABP4) in HepG2 cells (n = 6). (d) Western blot analysis of SREBP-1c, ACC1, FASN, CD36, FATP2 and FABP4 in HepG2 cells. β-actin was used as the internal standard (n = 3). (e) The schematic diagram of N-Me-PYs *in vivo* assay (n = 6). (f) Body weight changes during the 6 weeks experimental period (n = 6). (g) Representative histological results of H&E staining and oil red O staining of mice liver. Scale bar = 200 μm. The quantification of oil red O positive area was presented on the right (n = 6). (h-i) TG, TC and LDL-c levels in plasma (h) and liver homogenates (i) (n = 6).

We then utilized a human liver cell line HepG2 to clarify the impact of N-Me-6-PY and N-Me-4-PY on hepatosteatosis. CCK8 assay showed that the IC50 for N-Me-6-PY and N-Me-4-PY in HepG2 cells were 694.2 μM and 489.7 μM, respectively (Figure S3A). As previously reported, N-Me-6-PY is predominant in human and N-Me-4-PY in rodents,^31^ with the physiological plasma concentrations approximate to 1 μM and 0.5 μM, respectively.^31,32^ Thus, a gradient dose of N-Me-6-PY and N-Me-4-PY ranging from 2.5-fold to 10-fold of their physiological concentrations was used for *in vitro* intervention. Following stimulation with FFA, both N-Me-6-PY and N-Me-4-PY dose-dependently increased the burden of intracellular lipid droplets (Figures 3(a–b)) and raised intracellular TC and TG levels (Figure S3B) in hepatocytes. Furthermore, the mRNA expression levels of genes associated with de novo lipogenesis (SREBP-1c, ACC1, FASN) and fatty acid uptake (CD36, FATP2, FABP4) in the cells were upregulated following pretreatment with N-Me-6-PY and N-Me-4-PY (Figure 3(c)), while genes related to fatty acid oxidation (PPARα, CPT1α, ACOX1) and inflammation (TNFα, IL-6, IL-1β) showed no obvious alteration (Figure S3C). These findings were consistent at the protein level as well (Figures 3(d) and S3D).

Data are presented as the mean ± SEM and further analyzed by Welch’s ANOVA or one-way ANOVA with Fisher’s LSD post hoc test. #*P* < 0.05 in FFA vs vehicle. *P < 0.05; **P < 0.01; ***P < 0.001; ****P < 0.0001. N-Me-6-PY: N-Methyl-6-pyridone-3-carboxamide; N-Me-4-PY: N-Methyl-4-pyridone-5-carboxamide; FFA: free fatty acid; CD: chow diet; HFD: high fat diet; HYD: hydralazine; TG: total triglyceride; TC: total cholesterol; LDL-c: low-density lipoprotein cholesterol.

To further determine the pro-steatotic effect of N-Me-6-PY and N-Me-4-PY *in vivo*, five-to-six-week-old male C57BL/6J mice fed a CD or HFD were intraperitoneally injected with N-Me-PYs, a mixture of N-Me-6-PY (150 µg/kg) and N-Me-4-PY (75 µg/kg), or its vehicle for 6 weeks (Figure 3(e)). Moreover, HYD, a selective inhibitor of hepatic AOX1,^33,34^ was utilized to validate the therapeutic potential of AOX1 inhibition on NAFLD (Figure 3(e)). The administration of N-Me-PYs showed no obvious influence on body weight and liver weight (Figures 3(f) and S3E), yet mildly decreased the food intake of HFD mice (Figure S3F). As expected, supplementation with N-Me-PYs prominently aggravated hepatic lipid deposition in mice fed either CD or HFD (Figure 3(g)). Of note, both plasma and hepatic levels of TG, TC, and LDL-c, as well as plasma ALT were significantly increased in CD mice following N-Me-PYs administration (Figures 3(h–i), S3G). Similarly, plasma TC, LDL-c, and hepatic TG, LDL-c were apparently increased in HFD mice treated with N-Me-PYs, whereas the plasma TG, ALT, and hepatic TC were unaltered (Figures 3(h–i), S3G). In addition, the epididymal adipose tissue weight and adipocyte size were uninfluenced upon N-Me-PYs treatment (Figure S3H-I). These findings demonstrated that N-Me-PYs aggravated hepatic steatosis and contributed to lipid metabolic disturbance. Conversely, HYD effectively decreased body weight and liver weight (Figures 3(f) and S3E), mitigated hepatic lipid deposition and lipid metabolic disorder (Figures 3(g–i)), and improved the condition of epididymal adipose tissue (Figure S3H-I) in HFD mice, suggesting that inhibition of hepatic AOX1 might be a potential therapeutic strategy for NAFLD. Altogether, these results demonstrated that overaccumulation of N-Me-6-PY or N-Me-4-PY promoted de novo lipogenesis and fatty acid uptake capability in hepatocytes, thus aggravating hepatic steatosis.

### TRF reshapes gut microbiota in NAFLD mice

We further explored the mechanism by which TRF modulated AOX1 expression and reprogramed NAM metabolism. Previous studies have shown that TRF profoundly impacts gene expressions via realigning the circadian clock.^35,36^ However, the transcriptome landscape verified that the rhythmicity of liver AOX1 expression was not significantly altered under TRF in mammals,^35^ and proteome also exhibited relatively stable daily rhythms,^37^ suggesting the alteration of AOX1 expression by TRF might be independent of rhythm resynchronization. Previous studies have indicated that TRF can remodel the gut microbiota on both composition and metabolic function,^38,39^ thereby regulating host metabolic homeostasis. In addition, gut microbiota exhibits a modifiable effect on host gene regulation.^40–42^ To this, we hypothesized that gut microbiota might mediate the modulatory effects of TRF on host NAM metabolism, thus ameliorating hepatic steatosis.

**Figure 4.**
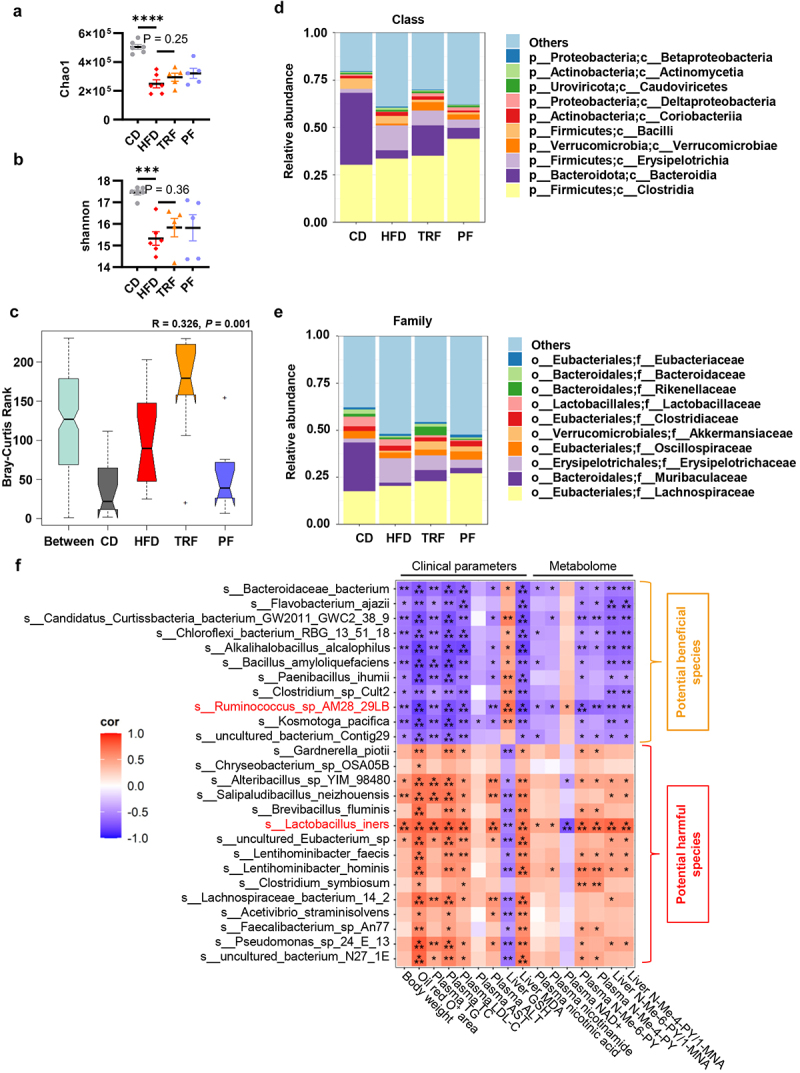
TRF reshapes gut microbiota in NAFLD mice. (a–b) Alpha-diversity of the gut microbiota among four groups indicated by Chao1 and Shannon index (*n* = 6 in CD and HFD, *n* = 5 in TRF and PF). (c) Principal coordinate analysis (PCoA) based on Bray–Curtis distance revealed β-diversity difference. (d) Relative abundances of gut microbiota at the class level. (e) Relative abundances of gut microbiota at the family level. (f) The heatmap showed the correlations between the abundance of differential species specifically regulated by TRF and various clinical parameters associated with NAFLD including body weight, oil red O staining positive area, plasma lipids, hepatic enzymes, oxidative indicators and metabolic data involving in NAM metabolism. The color scale represents the *Spearman’s* correlation coefficient.

Metagenome sequencing was performed on cecal contents obtained from the aforementioned mice. In terms of bacterial diversity, the α-diversity was obviously declined in HFD mice, while both TRF and PF displayed a trend of improvement, although statistically insignificant (Figures 4(a–b)). In addition, the principal coordinates analysis (PCoA) analysis based on Bray-Curtis distance demonstrated obvious differences in β-diversity among groups (Figure 4(c)). We also compared the gut microbiota composition profiles at different taxonomic levels. At the class level, the most predominant classes were Clostridia, Bacteroidia, Erysipelotrichia, Verrucomicrobiae, and Bacilli, and their relative abundance significantly differed among the four groups (Figure 4(d)). At the family level, Lachnospiraceae, Muribaculaceae, Erysipelotrichaceae, Oscillospiraceae, and Akkermansiaceae accounted for the largest proportion. Notably, TRF enriched the abundance of Akkermansiaceae and Rikenellaceae (Figure 4(e)). Previous studies have demonstrated that genera from these two families are less abundant in individuals with obesity, diabetes, and NAFLD,^43,44^ which is also associated with the improvement of HFD-induced metabolic disorders.^45,46^

Data are presented as the mean ± SEM and further analyzed by one-way ANOVA with Fisher’s LSD post hoc test. The correlation was tested by nonparametric Spearman’s correlation test. *P < 0.05; **P < 0.01; ***P < 0.001; ****P < 0.0001. CD: chow diet; HFD: high fat diet; TRF: time restricted feeding; PF: pair feeding. TG: total triglyceride; TC: total cholesterol; LDL-c: low-density lipoprotein cholesterol; ALT: alanine aminotransferase; AST: alanine aminotransferase; GSH: reduced glutathione; MDA: malondialdehyde; NA: nicotinic acid; NAM: nicotinamide; 1-MNA: 1-methylnicotinamide; N-Me-6-PY: N-Methyl-6-pyridone-3-carboxamide; N-Me-4-PY: N-Methyl-4-pyridone-5-carboxamide.

Next, we employed Metastats analysis^47^ to identify the bacterial species potentially involved in N-Me-6-PY and N-Me-4-PY production, as well as NAFLD progression. As the overlapping process shown in Figure S4A, a total of 26 bacterial species were searched out. The relative abundances of these species among the four groups were depicted in Figure S4B. Indeed, species such as Lactobacillus iners, Salipaludibacillus neizhouensis, Alteribacillus sp YIM-98480, uncultured Eubacterium sp, and Lachnospiraceae bacterium 14–2, which were downregulated following TRF, displayed apparently positive correlation with hepatic steatosis and lipid metabolic disturbance (Figure 4(f)). Conversely, Bacteroidaceae bacterium, Candidatus Curtissbacteria bacterium GW2011-GWC2-38-9, Alkalihalobacillus alcalophilus, Ruminococcus sp AM28-29LB, and Kosmotoga pacifica showed negative correlations with these indices. These species were depleted upon HFD, whereas they were restored by TRF intervention (Figure 4(f)). Importantly, Ruminococcus sp AM28-29LB and Lactobacillus iners exhibited the strongest correlation with the plasma levels of NAM catabolites, especially N-Me-6-PY, N-Me-4-PY, and NAD+, as well as the hepatic conversion ratios from 1-MNA to N-Me-6-PY and N-Me-4-PY (Figure 4(f)), suggested these bacterial species might potentially influence the hepatic NAM catabolism and the progression of NAFLD. Taken together, our findings implicated that the alteration of gut microbiota might mediate the regulatory effect of TRF on hepatic NAM catabolism, thereby ameliorating hepatic steatosis.

### Gut microbiota mediates the protective effect of TRF on NAFLD

**Figure 5.**
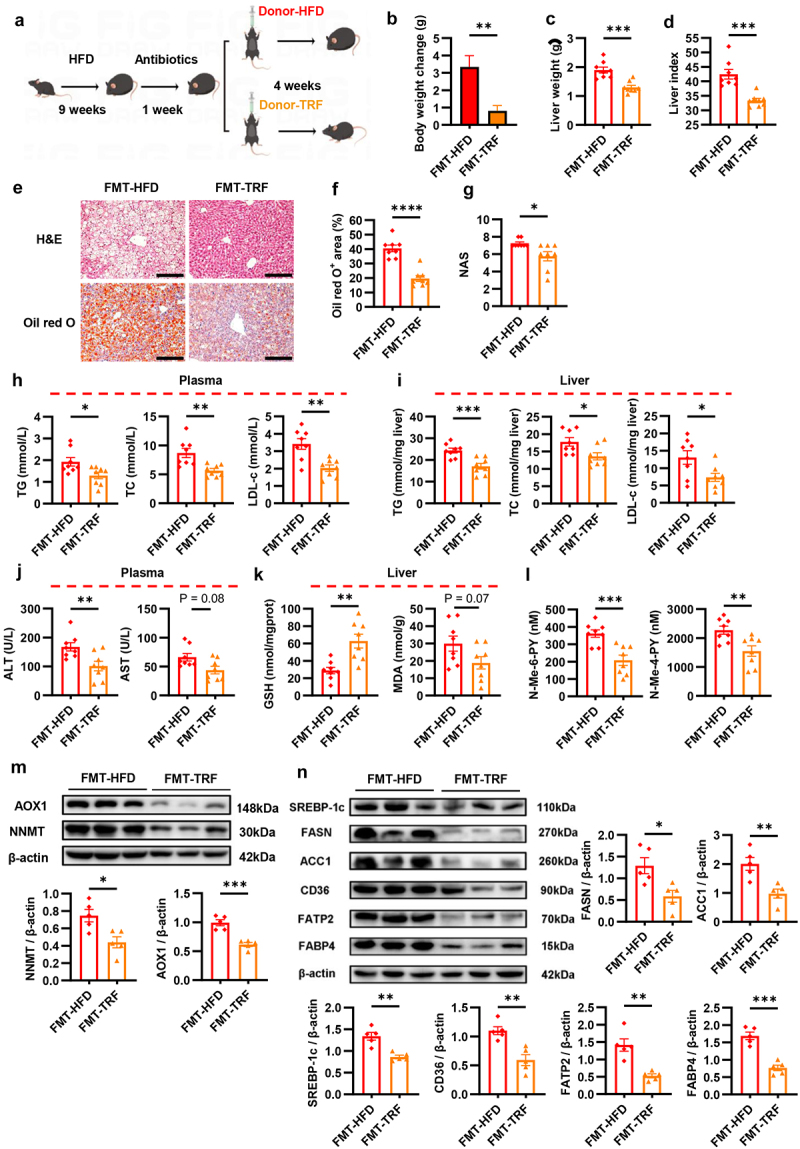
Gut microbiota mediates the protective effect of TRF on NAFLD. (a) The schematic diagram of FMT experiment design (n = 8). (b) The body weight changes following FMT (n = 8). (c) Mice liver weight (n = 8). (d) Mice liver index (n = 8). (e) Representative histological results of H&E staining and oil red O staining of mice liver. Scale bar = 200 μm. (f) The quantification of oil red O positive area (n = 8). (g) The quantification of NAS (n = 8). (H-I) TG, TC and LDL-c levels in plasma (h) and liver homogenates (i) (n = 8). (j) ALT and AST levels in plasma (n = 8). (k) GSH and MDA levels in liver homogenates (n = 8). (L) Plasma N-Me-6-PY and *N*-Me-4-PY levels (n = 8). (m) Western blot analysis of NNMT and AOX1 in mice liver. β-actin was used as the internal standard (n = 5). (n) Western blot analysis of SREBP-1c, ACC1, FASN, CD36, FATP2 and FABP4 in mice liver. β-actin was used as the internal standard (n = 5).

To probe into the causal relationship between TRF-mediated gut microbiota alteration and the improvement of NAFLD, an FMT experiment was conducted on HFD-induced obese mice. After a week of gut microbiota depletion using an antibiotic cocktail, mice were orally administered fecal bacterial suspensions from either HFD or TRF mice for another four consecutive weeks (three times per week) (Figure 5(a)). Consequently, mice transplanted with fecal microbiota from TRF donors exhibited a prominent decrease in body weight gain compared to those receiving FMT from HFD donors (Figure 5(b)). Surprisingly, TRF-dependent FMT led to pronounced reductions in liver weight, liver index, area of lipid deposition, and NAS (Figures 5(c–g) and S5A), along with the size of epididymal adipocytes (Figure S5B). In parallel, plasma lipids, liver enzymes, liver homogenate lipids, and hepatic oxidative stress levels were effectively improved after TRF-dependent FMT as well (Figures 5(h–k)). These findings further confirmed that gut microbiota, at least partially, mediates the protective effect of TRF pattern on hepatic steatosis and lipid metabolic disorder.

Data are presented as the mean ± SEM and further analyzed by unpaired t-test or Welch’s t-test. *P < 0.05; **P < 0.01; ***P < 0.001; ****P < 0.0001. FMT-HFD: recipients of feces from HFD group; FMT-TRF: recipients of feces from TRF group. FMT: fecal microbiota transplantation; NAS: NAFLD activity score; H&E: hematoxylin and eosin; TG: total triglyceride; TC: total cholesterol; LDL-c: low-density lipoprotein cholesterol; ALT: alanine aminotransferase; AST: alanine aminotransferase; GSH: reduced glutathione; MDA: malondialdehyde; N-Me-6-PY: N-Methyl-6-pyridone-3-carboxamide; N-Me-4-PY: N-Methyl-4-pyridone-5-carboxamide; NNMT: nicotinamide N-methyltransferase; AOX1: aldehyde oxidase 1.

Finally, we evaluated whether FMT can transfer the modulatory effect of TRF on NAM catabolism. Interestingly, mice received TRF fecal microbiota showed reductions of N-Me-6-PY and N-Me-4-PY in plasma by 43% and 32%, respectively (Figure 5(l)). Additionally, the protein expression levels of hepatic NNMT and AOX1 were declined by 41% and 38% (Figure 5(m)). Consistently, the gene expression level of hepatic AOX1 was also remarkably decreased (Figure S5C). Aligned with the *in vitro* findings, the reduction of N-Me-6-PY and N-Me-4-PY contributed to dramatic improvement in de novo lipogenesis and fatty acid uptake in the liver, as evidenced by the downregulation of FASN, ACC1, SREBP-1c, as well as CD36, FATP2, and FABP4 at both protein and gene expression levels (Figures 5(n) and S5D). Therefore, we concluded that gut microbiota mediated the metabolic modulatory effect of TRF, which suppressed the hyperactivation of NAM catabolism, and stabilized hepatic lipid metabolism function, thereby alleviating NAFLD.

## Discussion

**Figure 6.**
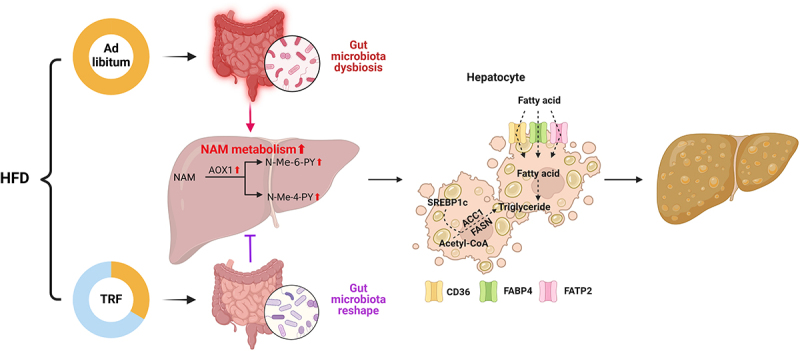
Global schema illustrating TRF ameliorates NAFLD through modulating host NAM metabolism via gut microbiota remodeling.

NAFLD has emerged as the most prevalent chronic liver disease worldwide, imposing a heavy economic burden. Given the lack of therapeutic agents, lifestyle interventions such as calorie restriction or intermittent fasting remain the mainstay of NAFLD treatment, which have been shown to improve liver biochemistry and hepatic steatosis.^48^ However, long-term adherence to caloric restriction or fasting in humans is challenging. Therefore, it is crucial to gain a deeper understanding of the mechanisms underlying the beneficial effects of dietary interventions on NAFLD in order to develop novel therapeutic targets. In concordance with previous studies,^49,50^ we confirmed the favorable NAFLD-protecting effects of TRF. Notably, the present study illustrated that TRF reprogrammed the disrupted hepatic NAM metabolism induced by HFD, which alleviated the overaccumulation of pro-steatotic metabolic products N-Me-6-PY and N-Me-4-PY, thereby suppressing the de novo lipogenesis and fatty acid uptake capability in hepatocytes. Furthermore, despite the fact that the concrete mechanism is still unclear, we provided evidence that transplantation of TRF-derived gut microbiota was sufficient to mimic the modulatory effect of TRF on the NAM metabolism, suggesting a gut microbiota-dependent manner (Figure 6).

HFD intake leads to gut microbiota dysbiosis, which subsequently augments hepatic NAM catabolism and contributes to the overaccumulation of N-Me-6-PY and N-Me-4-PY in plasma. N-Me-6-PY and N-Me-4-PY promote de novo lipogenesis and fatty acid uptake capacities in hepatocytes, ultimately aggravating NAFLD progression. Conversely, TRF intervention modulates NAM metabolism by reshaping the gut microbiota, thus declining plasma N-Me-6-PY and N-Me-4-PY and ameliorating NAFLD. The figure was established by Biorender.com.

HFD: high fat diet; TRF: time restricted feeding; NNMT: nicotinamide N-methyltransferase; AOX1: aldehyde oxidase 1; N-Me-6-PY: N-Methyl-6-pyridone-3-carboxamide; N-Me-4-PY: N-Methyl-4-pyridone-5-carboxamide; NAM: nicotinamide.

Using untargeted metabolomics to comprehensively evaluate the metabolic profile, we corroborated that TRF might alleviate NAFLD via modulating NAM metabolism. NAM has long been recognized for its metabolic benefits due to its ability of boosting the NAD+ level.^51,52^ In mammals, NAM is primarily metabolized in the liver through the catalytic actions of key enzymes, namely NNMT and AOX1, ultimately leading to the formation of N-Me-6-PY and N-Me-4-PY for urinary excretion.^53,54^ Recent studies announced that overactivation of NNMT contributes to the progression of liver steatosis,^55,56^ while inhibition of AOX1 decreased hepatic TG in NAFLD rats,^25^ both suggesting that dysregulation of NAM metabolism might engage in the pathological process of NAFLD. Interestingly, our data showed a dramatic increase in plasma N-Me-6-PY and N-Me-4-PY levels in mice under HFD. As the catabolic end products of NAM, N-Me-6-PY and N-Me-4-PY were previously characterized as uremic toxins that can reflect renal function.^57,58^ While elevated levels of N-Me-6-PY have been reported in patients with liver cirrhosis,^59^ a major complication of NAFLD, the causality, and underlying mechanisms remain completely unveiled. In the present study, we found that the circulating levels of N-Me-6-PY, as well as N-Me-4-PY, were positively correlated with various indices relating to hepatic steatosis, oxidative injury, and lipid metabolic disturbance. Moreover, supplementation of both N-Me-6-PY and N-Me-4-PY promoted de novo lipogenesis and fatty acid uptake *in vitro* and exacerbated hepatic lipid accumulation in mice fed either CD or HFD. Our findings, for the first time, shed light on the pro-steatotic effects and potential mechanisms of N-Me-6-PY and N-Me-4-PY. Notably, our study illustrated that the expression of hepatic AOX1, but not NNMT, was robustly increased in HFD mice. On the contrary, following TRF intervention, the expression of hepatic AOX1 was restored to normal levels, accompanied by a decline in circulating levels of N-Me-6-PY and N-Me-4-PY, ultimately ameliorating hepatic steatosis. Moreover, HYD, a potent hepatic AOX1 inhibitor, presented substantial alleviation effects on NAFLD. These data highlighted that inhibition of AOX1 might be a promising therapeutic strategy in the prevention of NAFLD.

The gut and liver are closely interconnected both physiologically and anatomically, and the gut–liver axis plays a vital role in bidirectional regulation during the development of NAFLD.^60,61^ Consistent with previous reports,^23,62^ our data revealed that TRF partially restored the gut microbiota dysbiosis community induced by HFD. Notably, we found the potential beneficial species enriched in TRF mice, Ruminococcus sp AM28-29LB, exhibited the strongest negative correlation with the parameters related to NAFLD and host NAM catabolism. Previous studies indicated that the abundances of Ruminococcus spp. were lower in patients with NAFLD in comparison to healthy individuals,^63^ while administration of Ruminococcus spp. protected against liver damage in a NAFLD murine model.^64^ Moreover, Ruminococcus spp. had been characterized as a vitamin B3 (NA and NAM)-producing bacterium,^65^ which may potentially affect the NAM metabolism. Conversely, among the species enriched in HFD mice, Lactobacillus iners exhibited the most positive association with the severity of NAFLD, as well as the augmenting NAM catabolism. Lactobacillus iners was initially recognized as a prevalent species in the vaginal niche.^66^ However, recent studies have shown a remarkable upregulation trend in patients with NAFLD,^67^ as well as rheumatoid arthritis.^68^ In addition, Lactobacillus iners could be detected in various types of cancer which altered tumor metabolism and led to therapeutic resistance.^69^ These observations suggest that Lactobacillus iners might be a potential conditional pathogenic gut microbe that is associated with human diseases. Collectively, although the causal relationship remains uncertain, our study, along with previous publications, highly suggests a potential linkage between the alteration of the gut microbiota community and host NAM metabolism. Our findings are supported by the fact that the trans-kingdom cooperation between bacteria and mammalian cells in the host NAM metabolism.^70^ In agreement with this, transplantation of TRF-derived fecal microbiota strikingly diminished the plasma levels of N-Me-6-PY and N-Me-4-PY, leading to a decline in hepatic lipogenesis and fatty acid uptake. In fact, gut microbiota can regulate host gene expression by remodeling host chromatin,^71^ prompting differential splicing,^72^ altering the epigenetic landscape,^73^ or influencing host transcription.^74^ The present study illustrated that the expressions of hepatic AOX1 were downregulated following transplantation of TRF-derived fecal microbiota, which essentially recapitulated the modulatory effect of TRF intervention, suggesting that gut microbiota might act as an intermediate factor bridging TRF regimen and host NAM metabolism. Taken together, our study uncovered novel insights into the regulatory roles of gut microbiota in TRF-mediated protection of NAFLD.

Indeed, our study has several limitations that require further investigation. Firstly, our study clarified N-Me-6-PY and N-Me-4-PY increased lipogenesis and fatty acid uptake in hepatocytes, which served as pathogenic factors in the progression of NAFLD, but their direct target remained poorly characterized. Additionally, although we have provided evidence that the modulation of the gut microbiota plays an important role in mediating the beneficial effects of TRF on hepatic NAM metabolism, as supported by correlation analysis and FMT experiment. However, further analysis is necessary to elucidate the detailed species, genes, or enzymes within the gut microbiota that are responsible for mediating this process. Then, only male animals were utilized in the present study; however, the potential presence of sex-specific disparities necessitates further elucidation.

## Conclusion

In summary, our study revealed that the TRF regimen modulated the host NAM metabolic homeostasis via gut microbiota reshaping, which mediated its protective effects on NAFLD. Our findings provide a promising strategy for the prevention and treatment of NAFLD.

## Supplementary Material

Supplemental Material

## Data Availability

The untargeted plasma metabolomic and gut microbial metagenome sequencing data are both available on the Dryad database under doi:10.5061/dryad.83bk3j9zz.
