# Supplementary material for: Time-restricted feeding ameliorates non-alcoholic fatty liver disease through modulating hepatic nicotinamide metabolism via gut microbiota remodeling
Source: Gut Microbes. 2024 Aug 18;16(1):2390164. doi: 10.1080/19490976.2024.2390164 (PMC11332628; doi:10.1080/19490976.2024.2390164)
Supplement: Supplemental Material [file KGMI_A_2390164_SM1047.zip › supplementary file/rael-2023-0794-File002.docx]

# Appendix A

For robustness checking, we added three regressions: baseline regression excluding year-firm fixed effect (Panel A of Table A1), baseline regression excluding control variables (Panel B of Table A1), and regression of Bloomberg’s ESG rating on pay dispersion (Panel C of Table A1). And the conclusions are consistent with the baseline regression.

Table A1. Robustness checks

| **Panel A. Baseline regression excluding year-firm fixed effect** | | | | | |
| --- | --- | --- | --- | --- | --- |
|  | Dependent Variable: *SNSI ESG rating* | | | | |
|  | (1) | (2) | (3) | (4) | (5) |
| $\text{Vertical\_PD}_{\text{firm}}$ | 0.381*** |  |  |  |  |
|  | (14.59) |  |  |  |  |
| $\text{Vertical\_PD}_{\text{man}}$ |  | 0.190*** |  |  |  |
|  |  | (8.53) |  |  |  |
| $\text{Horizontal\_PD}_{\text{dir}}$ |  |  | -0.042*** |  |  |
|  |  |  | (-3.64) |  |  |
| $\text{Horizontal\_PD}_{\text{sup}}$ |  |  |  | -0.083*** |  |
|  |  |  |  | (-2.80) |  |
| $\text{Horizontal\_PD}_{\text{exe}}$ |  |  |  |  | -0.109*** |
|  |  |  |  |  | (-4.37) |
| Observations | 30,355 | 30,355 | 30,355 | 30,355 | 30,355 |
| Control variables | YES | YES | YES | YES | YES |
| **Panel B. Baseline regression excluding control variables** | | | | | |
|  | Dependent Variable: *SNSI ESG rating* | | | | |
|  | (1) | (2) | (3) | (4) | (5) |
| $\text{Vertical\_PD}_{\text{firm}}$ | 0.329*** |  |  |  |  |
|  | (10.66) |  |  |  |  |
| $\text{Vertical\_PD}_{\text{man}}$ |  | 0.166*** |  |  |  |
|  |  | (6.77) |  |  |  |
| $\text{Horizontal\_PD}_{\text{dir}}$ |  |  | -0.027** |  |  |
|  |  |  | (-2.17) |  |  |
| $\text{Horizontal\_PD}_{\text{sup}}$ |  |  |  | -0.064** |  |
|  |  |  |  | (-2.04) |  |
| $\text{Horizontal\_PD}_{\text{exe}}$ |  |  |  |  | -0.083*** |
|  |  |  |  |  | (-3.15) |
| Observations | 30,355 | 30,355 | 30,355 | 30,355 | 30,355 |
| Company FE | YES | YES | YES | YES | YES |
| Year FE | YES | YES | YES | YES | YES |
| **Panel C. Regression of Bloomberg’s ESG rating on pay dispersion** | | | | | |
|  | Dependent Variable: *Bloomberg ESG rating* | | | | |
|  | (1) | (2) | (3) | (4) | (5) |
| $\text{Vertical\_PD}_{\text{firm}}$ | 2.499* |  |  |  |  |
|  | (1.92) |  |  |  |  |
| $\text{Vertical\_PD}_{\text{man}}$ |  | 5.818*** |  |  |  |
|  |  | (3.16) |  |  |  |
| $\text{Horizontal\_PD}_{\text{dir}}$ |  |  | -1.393** |  |  |
|  |  |  | (-2.14) |  |  |
| $\text{Horizontal\_PD}_{\text{sup}}$ |  |  |  | -5.836*** |  |
|  |  |  |  | (-2.67) |  |
| $\text{Horizontal\_PD}_{\text{exe}}$ |  |  |  |  | -3.668** |
|  |  |  |  |  | (-2.24) |
| Observations | 10,337 | 10,337 | 10,337 | 10,337 | 10,337 |
| Company FE | YES | YES | YES | YES | YES |
| Year FE | YES | YES | YES | YES | YES |
| Control variables | YES | YES | YES | YES | YES |
| Note: t-statistics in parentheses. *** means p<0.01, ** means p<0.05, * means p<0.1. | | | | | |

To address the potential endogeneity problems caused by reverse causality, we add Two-Stage least squares (2SLS) regression. We use 2 instrument variables: the number of VPs and the median pay dispersion for firms in the same industry in the same year. These 2 variables affect firm’s pay dispersion, but won’t impact firm’s ESG score directly. The results in Panel A of Table A2 verify the robustness of baseline results by addressing endogeneity. The weak instrumental variable test and overidentifying test have been done. GMM regression is also used to address endogeneity, and the result is consistent (Panel B of Table A2).

Table A2. Endogeneity treatment

| **Panel A. Two-Stage least squares (2SLS) regression** | | | | | |
| --- | --- | --- | --- | --- | --- |
|  | Dependent Variable: *SNSI ESG rating* | | | | |
|  | (1) | (2) | (3) | (4) | (5) |
| $\text{Vertical\_PD}_{\text{firm}}$ | 0.399** |  |  |  |  |
|  | (2.33) |  |  |  |  |
| $\text{Vertical\_PD}_{\text{man}}$ |  | 0.578** |  |  |  |
|  |  | (2.39) |  |  |  |
| $\text{Horizontal\_PD}_{\text{dir}}$ |  |  | -0.396*** |  |  |
|  |  |  | (-4.62) |  |  |
| $\text{Horizontal\_PD}_{\text{sup}}$ |  |  |  | -0.877*** |  |
|  |  |  |  | (-3.51) |  |
| $\text{Horizontal\_PD}_{\text{exe}}$ |  |  |  |  | -0.831*** |
|  |  |  |  |  | (-5.56) |
| **Panel B. GMM regression** | | | | | |
|  | Dependent Variable: *SNSI ESG rating* | | | | |
|  | (1) | (2) | (3) | (4) | (5) |
| $\text{Vertical\_PD}_{\text{firm}}$ | 0.386*** |  |  |  |  |
|  | (8.85) |  |  |  |  |
| $\text{Vertical\_PD}_{\text{man}}$ |  | 0.405*** |  |  |  |
|  |  | (7.61) |  |  |  |
| $\text{Horizontal\_PD}_{\text{dir}}$ |  |  | -0.146*** |  |  |
|  |  |  | (-3.40) |  |  |
| $\text{Horizontal\_PD}_{\text{sup}}$ |  |  |  | -0.433*** |  |
|  |  |  |  | (-3.52) |  |
| $\text{Horizontal\_PD}_{\text{exe}}$ |  |  |  |  | -0.585*** |
|  |  |  |  |  | (-5.29) |
| Observations | 30,355 | 30,355 | 30,355 | 30,355 | 30,355 |
| Company FE | YES | YES | YES | YES | YES |
| Year FE | YES | YES | YES | YES | YES |
| Control variables | YES | YES | YES | YES | YES |
| Note: z-statistics in parentheses in panel A; t-statistics in parentheses in panel B. The pay dispersion with a lag of 2 to 5 periods is used as instrument variables during GMM estimating. *** means p<0.01, ** means p<0.05, * means p<0.1. | | | | | |

# Appendix B

We further investigate the relationship between pay dispersion and the individual categories of ESG to show more details. Intuitively, the pay dispersion should have significant influence on the Social and Governance aspects. The results find the two vertical pay dispersions ($\text{Vertical\_PD}_{\text{firm}}$ and $\text{Vertical\_PD}_{\text{man}}$) both have the biggest positive influence on the Social score of ESG ($\beta=$2.339*** and $\beta=$2.153***); the horizontal pay dispersion of directors has the biggest negative influence on the Social score ($\beta=-$0.454***); and the horizontal pay dispersion of executives has the biggest negative influence on the Governance score ($\beta=-$0.584***). Meanwhile, we find the pay dispersion will affect firm’s Environmental performance (in Panel A). This result is complementary to Zhang et al. (2020), who also found the corporate environmental responsibility is related to the pay dispersion.

Table B1. Regressions of E, S, and G scores on pay dispersion

| **Panel A.** Dependent Variable: *Environmental (E* *score)* | | | | | |
| --- | --- | --- | --- | --- | --- |
|  | (1) | (2) | (3) | (4) | (5) |
| $\text{Vertical\_PD}_{\text{firm}}$ | 0.920*** |  |  |  |  |
|  | (5.09) |  |  |  |  |
| $\text{Vertical\_PD}_{\text{man}}$ |  | 0.333 |  |  |  |
|  |  | (1.52) |  |  |  |
| $\text{Horizontal\_PD}_{\text{dir}}$ |  |  | -0.074 |  |  |
|  |  |  | (-1.04) |  |  |
| $\text{Horizontal\_PD}_{\text{sup}}$ |  |  |  | -0.686*** |  |
|  |  |  |  | (-3.55) |  |
| $\text{Horizontal\_PD}_{\text{exe}}$ |  |  |  |  | -0.547*** |
|  |  |  |  |  | (-3.58) |
| **Panel B.** Dependent Variable: *Social (S* *score)* | | | | | |
|  | (1) | (2) | (3) | (4) | (5) |
| $\text{Vertical\_PD}_{\text{firm}}$ | 2.339*** |  |  |  |  |
|  | (8.32) |  |  |  |  |
| $\text{Vertical\_PD}_{\text{man}}$ |  | 2.153*** |  |  |  |
|  |  | (6.32) |  |  |  |
| $\text{Horizontal\_PD}_{\text{dir}}$ |  |  | -0.454*** |  |  |
|  |  |  | (-4.11) |  |  |
| $\text{Horizontal\_PD}_{\text{sup}}$ |  |  |  | -0.018 |  |
|  |  |  |  | (-0.06) |  |
| $\text{Horizontal\_PD}_{\text{exe}}$ |  |  |  |  | -0.253 |
|  |  |  |  |  | (-1.06) |
| **Panel C.** Dependent Variable: *Governance (G* *score)* | | | | | |
|  | (1) | (2) | (3) | (4) | (5) |
| $\text{Vertical\_PD}_{\text{firm}}$ | 1.290*** |  |  |  |  |
|  | (5.80) |  |  |  |  |
| $\text{Vertical\_PD}_{\text{man}}$ |  | 1.582*** |  |  |  |
|  |  | (5.88) |  |  |  |
| $\text{Horizontal\_PD}_{\text{dir}}$ |  |  | 0.089 |  |  |
|  |  |  | (1.02) |  |  |
| $\text{Horizontal\_PD}_{\text{sup}}$ |  |  |  | -0.388 |  |
|  |  |  |  | (-1.63) |  |
| $\text{Horizontal\_PD}_{\text{exe}}$ |  |  |  |  | -0.584*** |
|  |  |  |  |  | (-3.10) |
| Observations | 30,355 | 30,355 | 30,355 | 30,355 | 30,355 |
| Company FE | YES | YES | YES | YES | YES |
| Year FE | YES | YES | YES | YES | YES |
| Control variables | YES | YES | YES | YES | YES |

Note: t-statistics in parentheses; *** means p<0.01, ** means p<0.05, * means p<0.1.

# Appendix C

Table C1. Correlation coefficients of major variables

|  | *ESG rating* | *Vertical_PD_firm_* | *Vertical_PD_man_* | *Horizontal_PD_dir_* | *Horizontal_PD_sup_* | *Horizontal_PD_exe_* | *FS* | *FA* | *FAL* | *FTA* | *FLS* | *FFA* | *FSN* |
| --- | --- | --- | --- | --- | --- | --- | --- | --- | --- | --- | --- | --- | --- |
| *ESG rating* | 1 |  |  |  |  |  |  |  |  |  |  |  |  |
| *Vertical_PD_firm_* | 0.1875 | 1 |  |  |  |  |  |  |  |  |  |  |  |
| *Vertical_PD_man_* | 0.137 | 0.8175 | 1 |  |  |  |  |  |  |  |  |  |  |
| *Horizontal_PD_dir_* | -0.0202 | 0.0428 | 0.1373 | 1 |  |  |  |  |  |  |  |  |  |
| *Horizontal_PD_sup_* | -0.0471 | 0.0828 | 0.1254 | 0.2985 | 1 |  |  |  |  |  |  |  |  |
| *Horizontal_PD_exe_* | -0.0711 | 0.0708 | 0.1125 | 0.212 | 0.7464 | 1 |  |  |  |  |  |  |  |
| *FS* | 0.1246 | 0.2236 | 0.2137 | 0.0161 | -0.0513 | -0.0486 | 1 |  |  |  |  |  |  |
| *FA* | -0.0204 | 0.2093 | 0.1806 | 0.0721 | 0.0224 | 0.0332 | 0.0292 | 1 |  |  |  |  |  |
| *FAL* | -0.0509 | 0.0351 | 0.0342 | 0.0346 | -0.0224 | -0.015 | 0.0651 | 0.0643 | 1 |  |  |  |  |
| *FTA* | 0.0055 | 0.0051 | 0.0034 | -0.0019 | 0.0295 | 0.0241 | -0.0061 | -0.0388 | -0.007 | 1 |  |  |  |
| *FLS* | 0.0969 | 0.0145 | 0.0154 | 0.0924 | -0.0445 | -0.0514 | 0.1407 | -0.1045 | 0.0155 | -0.0216 | 1 |  |  |
| *FFA* | 0.092 | 0.1126 | 0.0894 | 0.0043 | 0.0085 | 0.0145 | 0.0176 | 0.0187 | -0.0354 | 0.0846 | 0.0445 | 1 |  |
| *FSN* | 0.0824 | 0.0382 | 0.0596 | 0.2181 | -0.1961 | -0.2007 | 0.1372 | 0.145 | 0.109 | -0.0709 | 0.2219 | -0.0376 | 1 |
