# Supplementary figures and images for: Time-restricted feeding ameliorates non-alcoholic fatty liver disease through modulating hepatic nicotinamide metabolism via gut microbiota remodeling
Source: Gut Microbes. 2024 Aug 18;16(1):2390164. doi: 10.1080/19490976.2024.2390164 (PMC11332628; doi:10.1080/19490976.2024.2390164)

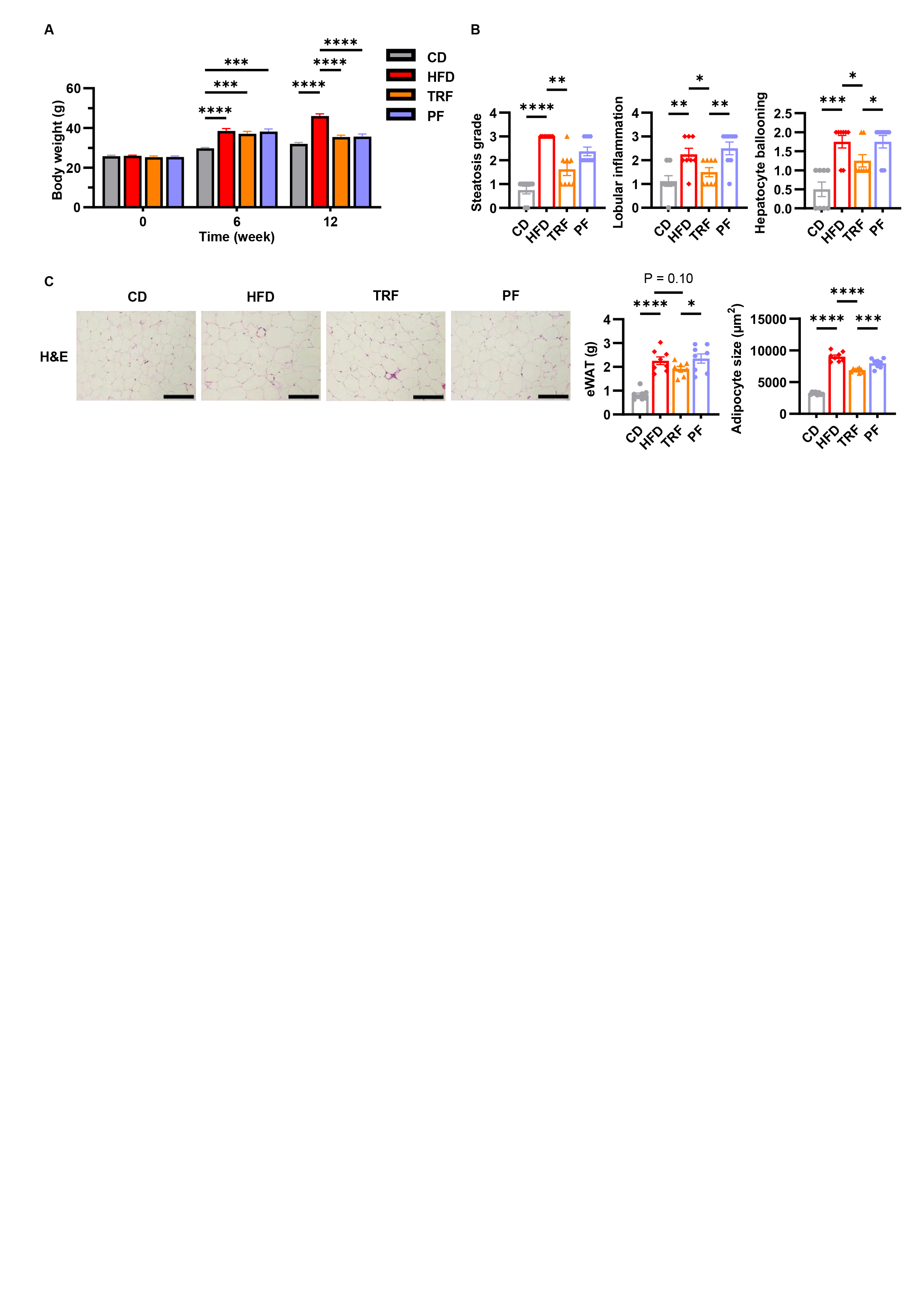

Supplement: Supplemental Material [file KGMI_A_2390164_SM1047.zip › supplementary file/Figure S1.jpg]

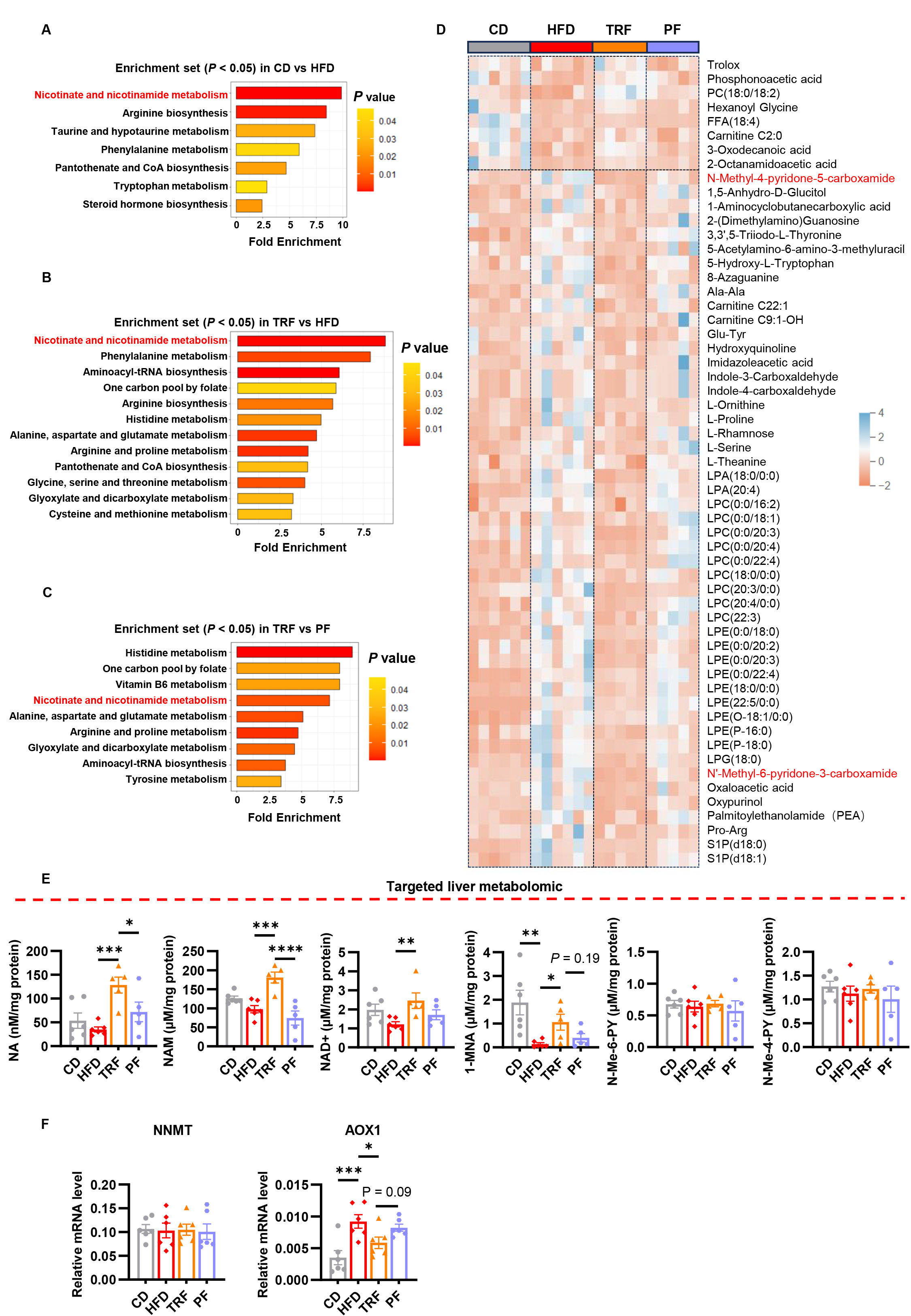

Supplement: Supplemental Material [file KGMI_A_2390164_SM1047.zip › supplementary file/Figure S2.jpg]

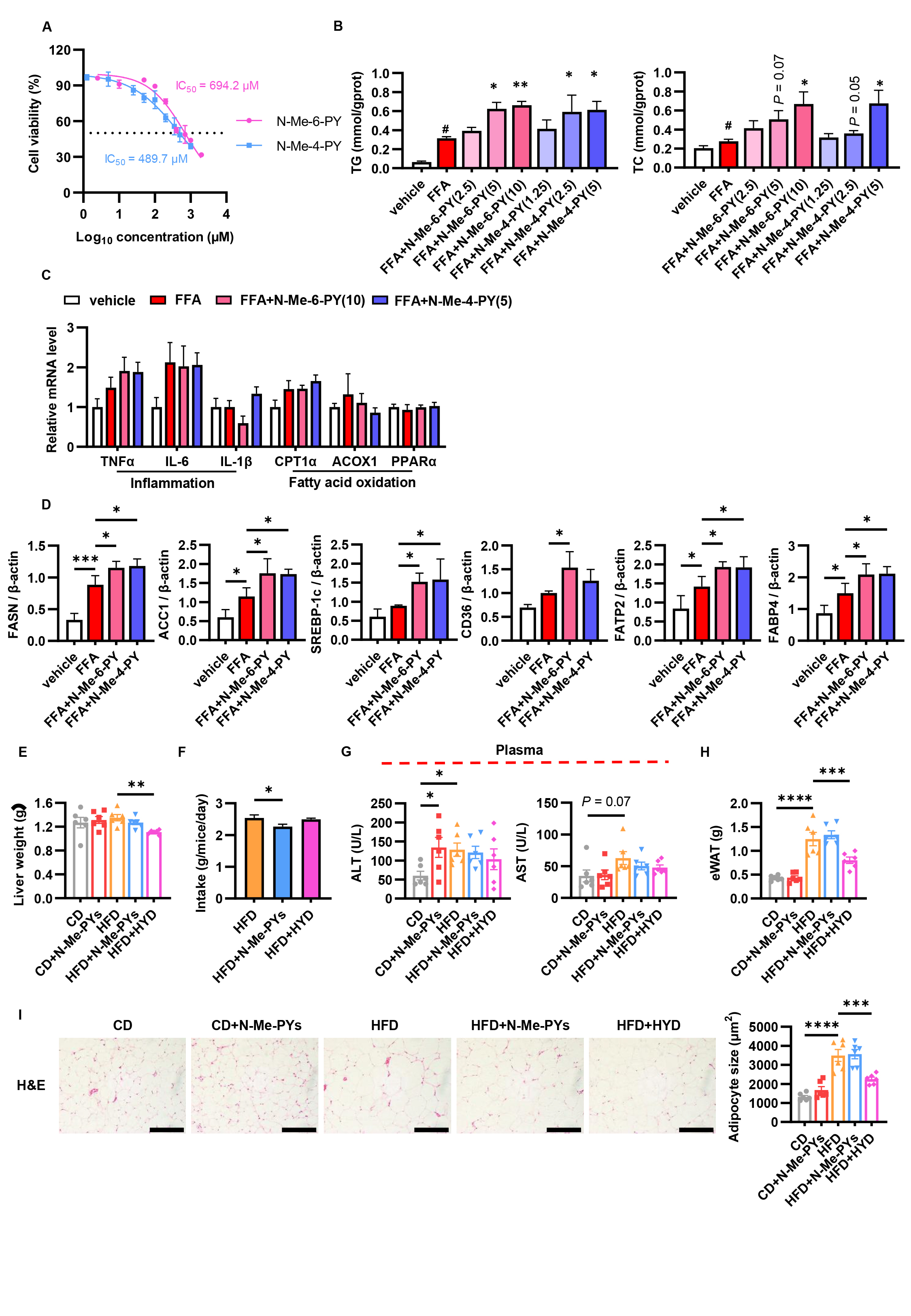

Supplement: Supplemental Material [file KGMI_A_2390164_SM1047.zip › supplementary file/Figure S3.jpg]

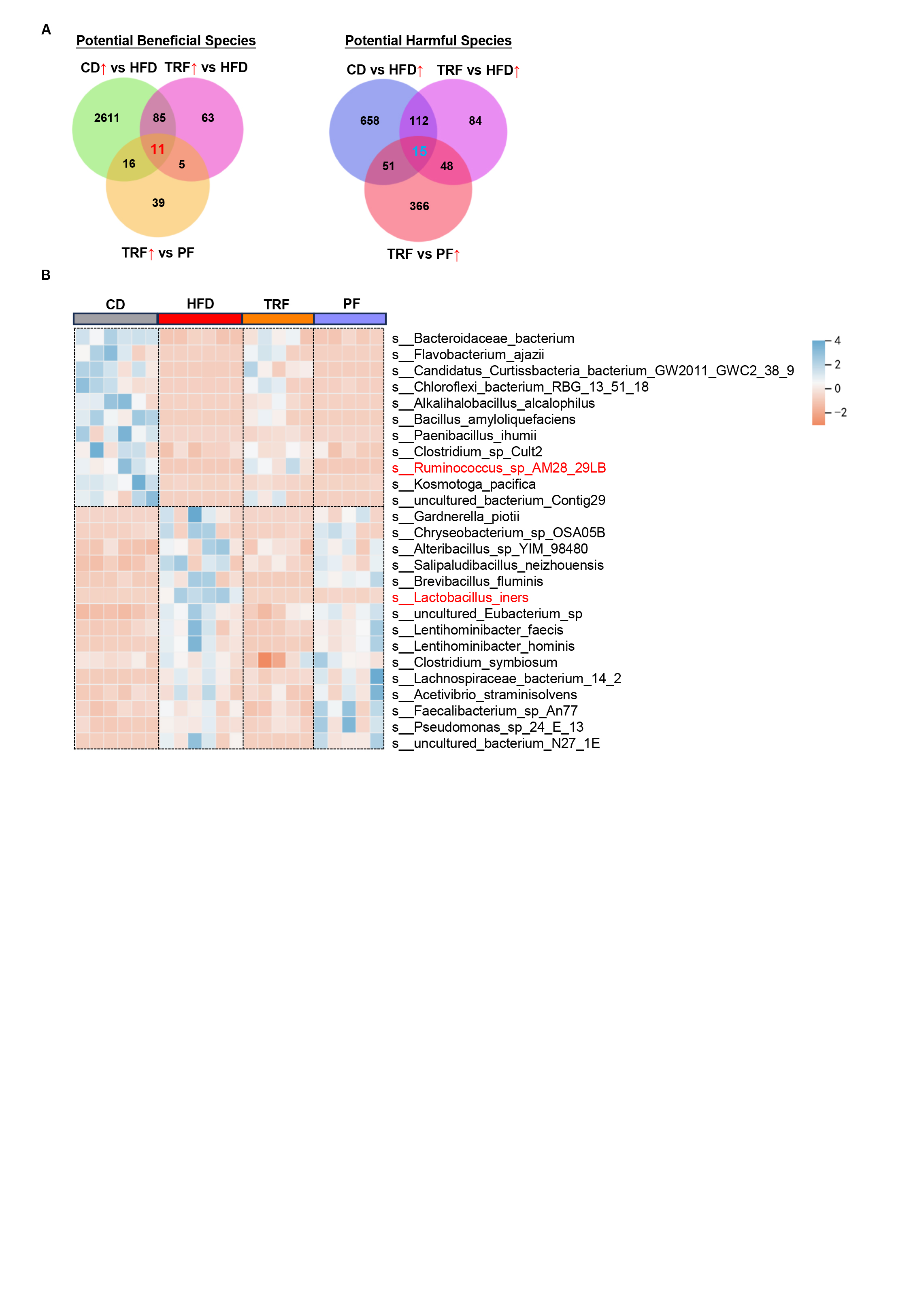

Supplement: Supplemental Material [file KGMI_A_2390164_SM1047.zip › supplementary file/Figure S4.jpg]

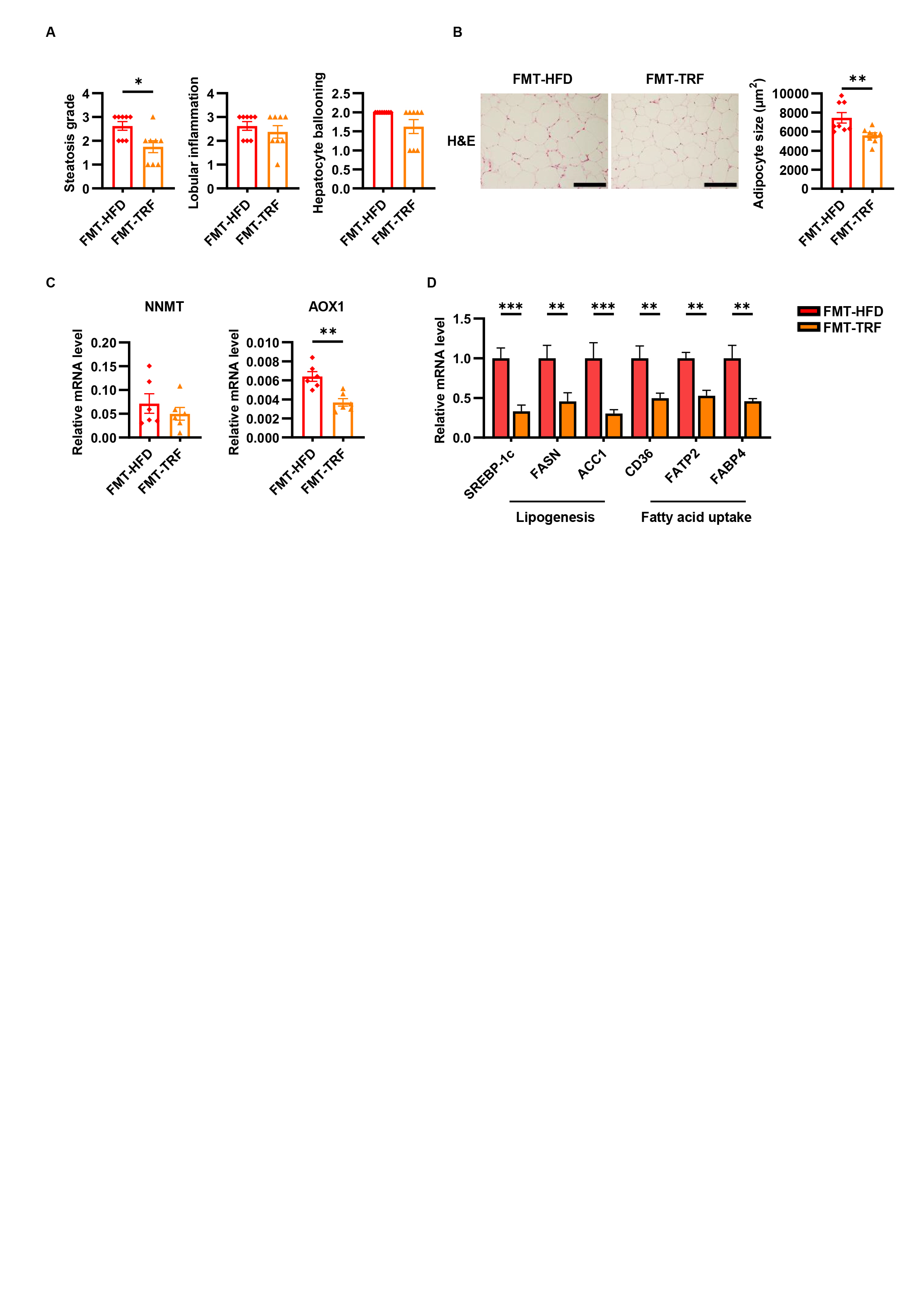

Supplement: Supplemental Material [file KGMI_A_2390164_SM1047.zip › supplementary file/Figure S5.jpg]
